# Analyzing synchronized clusters in neuron networks

**DOI:** 10.1038/s41598-020-73269-9

**Published:** 2020-10-01

**Authors:** Matteo Lodi, Fabio Della Rossa, Francesco Sorrentino, Marco Storace

**Affiliations:** 1 DITEN, University of Genoa, Via Opera Pia 11a, 16145 Genova, Italy; 2 Mechanical Engineering Department, University of New Mexico, Albuquerque, NM 87131 USA; 3 Dipartimento di Elettronica, Informazione e Bioingegneria, Politecnico di Milano, 20133 Milan, Italy

**Keywords:** Applied mathematics, Mathematics and computing

## Abstract

The presence of synchronized clusters in neuron networks is a hallmark of information transmission and processing. Common approaches to study cluster synchronization in networks of coupled oscillators ground on simplifying assumptions, which often neglect key biological features of neuron networks. Here we propose a general framework to study presence and stability of synchronous clusters in more realistic models of neuron networks, characterized by the presence of delays, different kinds of neurons and synapses. Application of this framework to two examples with different size and features (the directed network of the macaque cerebral cortex and the swim central pattern generator of a mollusc) provides an interpretation key to explain known functional mechanisms emerging from the combination of anatomy and neuron dynamics. The cluster synchronization analysis is carried out also by changing parameters and studying bifurcations. Despite some modeling simplifications in one of the examples, the obtained results are in good agreement with previously reported biological data.

## Introduction

Understanding the functional mechanisms of a given system/phenomenon and describing it through mathematical equations as simple as possible (according to the *Occam’s razor* principle) is the Holy Grail of modeling. Among the others, neuron networks are the object of many studies due to their complex behaviors; understanding the functional mechanisms of information transmission and processing in this kind of networks is one of the most difficult and fascinating challenges faced by the scientific community, at the crossroad between many disciplines.

The level of abstraction used to describe neuron networks can significantly change according to the modeling goals, complexity of the network to be modeled and background knowledge^1^. Consequently, the basic elements of the nervous system (neurons and synapses) are modeled by trading off accuracy and complexity^2^. Neurons in the same network can be of different kinds and their synaptic connections, also of different kinds, can be either electrical or chemical, either excitatory or inhibitory, either directed or undirected, and may transmit signals with different delays. In this paper, we focus on deterministic models of these networks.

A commonly observed phenomenon in networks of neurons is the formation of *synchronous clusters*, i.e., groups of neurons that fulfill some synchrony conditions^3–5^, usually expressed in terms of temporal correlation between neural signals. These clusters are strongly related to information transmission and processing^6^. Living Nature is quite far from determinism, with unavoidable differences arising due to the presence of uncertainty/noise in any measured quantity (variables and parameters); therefore, instead of exact clustering, slightly imperfect clusters will be observed in any real experiment. This notwithstanding, recent efforts have been devoted to apply nonlinear dynamics concepts and network theory to the neuroscience context^1,7^. This is done by resorting to deterministic models (which is a first-order simplification) and studying the presence and the stability of synchronized clusters in networks based on one or more assumptions (second-order simplifications), such as identical neurons/synapses, weak interactions, absence of delays, or undirected/diffusive connections. As an example, the phase response curve (PRC) theory^8,9^ (grounded on the assumption of weak interactions) is often used to study both clustering in networks of (weakly coupled) generic oscillators and how two-cluster solutions and global synchrony arise through bifurcations in networks of neurons^10,11^. In this paper we propose a variational method that can be applied to characterize stability of the cluster synchronous solution, when some of the mentioned second-order simplifications are lifted. The proposed method allows finding better approximations to more realistic (i.e., not exactly synchronized) solutions and it provides understanding of basic cluster synchronization mechanisms, whose robustness can be checked by resorting to other less deterministic approaches.

On the whole, the method (based on the multi-layer network formalism) can be used to analyze exact cluster synchronization (CS) in neuron networks with directed connections, delays, couplings that depend on both the presynaptic and the postsynaptic neurons, and different kinds of nodes and synapses. The main novelty is the generalization to this general framework of a stability analysis method previously developed for a tighter class of networks^12–20^. Our goal is to achieve improved understanding of the causal influence that each network element exerts on the other elements, thus shedding light on how functions emerge from structural connectivity, combined with neuronal dynamics. We successfully apply our approach to two neuron networks on different scales: the first one is the small-scale central pattern generator responsible for swim motion of the nudibranch mollusc *Dendronotus iris*; the second one is the large-scale cortical connectivity network of the macaque, which describes anatomical connections among different cortical areas. In both cases, the analysis is carried out also changing some significant network parameters (following real experiments that we use as benchmarks), by exploiting bifurcation analysis combined with the proposed CS analysis. The obtained results are in agreement with previously reported biological behaviors for both case studies, indicating that the proposed analysis can be useful to study real neuron networks, to predict the existence of stable synchronous clusters, and to perform virtual experiments in view of better focused real experiments.

## Results

### Network model

The networks described in the Introduction can be modeled by the following set of dynamical equations, describing a multi-layer network^21^, ($i = 1,\ldots ,N$)

1 $$\begin{aligned} \dot{x}_i = {\tilde{f}}_i(x_i(t)) + \sum _{k=1}^{L}\sigma ^k \sum _{j=1}^{N} A_{ij}^k h^k(x_i(t),x_j(t-\delta _k)), \end{aligned}$$

where $x_i \in {\mathbb {R}}^n$ is the *n*-dimensional state vector of the *i*-th neuron, ${\tilde{f}}_i : {\mathbb {R}}^n \rightarrow {\mathbb {R}}^n$ is the vector field of the isolated *i*-th neuron, $\sigma ^k \in {\mathbb {R}}$ is the coupling strength of the *k*-th kind of link, $A^k$ is the possibly weighted and directed coupling matrix (or adjacency matrix) that describes the connectivity of the network with respect to the *k*-th kind of link, for which the interaction between two generic cells *i* and *j* is described by the nonlinear function $h^k: {\mathbb {R}}^n \times {\mathbb {R}}^n \rightarrow {\mathbb {R}}^n$, and $\delta _k$ is the axon transmission delay characteristic of the *k*-th kind of link. For example, electrical synapses (gap junctions) are almost instantaneous, whereas the delay associated with transmission of a signal through a chemical synapse may be considerably longer.

A neuron model is described by a state vector $x_i$, whose first component $V_i$ typically represents the membrane potential of the neuron. A synapse model can either neglect or include the neurotransmitter dynamics, therefore we can have instantaneous or dynamical synapses, respectively. In both cases, we assume that the synaptic coupling influences only the dynamics of $V_i$ and not of the other state variables contained in $x_i$: therefore, the first component of the vector $h^k(\cdot )$ is a scalar function (called *activation function*) $a^k(V_i(t),x_j(t-\delta _k))$ and the remaining components are null. For instantaneous synapses, the activation depends on the membrane potential of the pre- and post-synaptic neurons, therefore it can be expressed as $a^k(V_i(t),V_j(t-\delta _k))$. By contrast, for dynamical synapses the activation $a^k$ is a function of a state variable $s_j^k$ (in addition to $V_i$), whose dynamics usually depends on the pre-synaptic membrane potential $V_j$ (see Sect. 1 in the Supplementary Information for an example). For this reason, all dynamical synapses of kind *k* connecting the neuron *j* with other neurons share the same state $s_j^k$, which can be added to vector $x_j$.

We further assume each individual node can be of one out of *M* different types (with $M \le N$): ${\tilde{f}}_i(x) = {\tilde{f}}_j(x)$ if *i* and *j* are of the same type, ${\tilde{f}}_i(x) \ne {\tilde{f}}_j(x)$ otherwise. Often, the difference (physical or functional) between two types of neurons is accounted for through a different value of one or more model parameters. Within this general framework, where all oscillators can be different, if $M<< N$ the vector fields ${\tilde{f}}_i$ are not all different, but belong to a restricted set of *M* models. Assuming that all node states share the same dimension *n* is not restrictive: in the case of state vectors $x_i$ with different dimensions $n_i$, it is sufficient to define ${n = \max _{i} n_i}$ and set to 0 the components in excess.

Different from most models introduced in the literature, the set of equations (1) accounts for the following realistic properties of neuron networks: (i) each synapse depends (algebraically in the case of instantaneous/fast synapses or dynamically in the case of slower synapses) on the state of both the pre-synaptic and the post-synaptic neuron, (ii) each synapse between two neurons is in general a direct connection that can be of different kinds (such as either chemical inhibitory/excitatory or electrical excitatory), and (iii) the transmission of information along synapses can be non-instantaneous, which may be due in part to local synaptic filtering of exchanged spikes, and in part to the distribution of the axonal transmission delays^22^. We wish to emphasize that current methods developed to analyze CS in complex networks^15,20^ are unable to handle features (i), (ii) and (iii) above.

**Figure 1:**
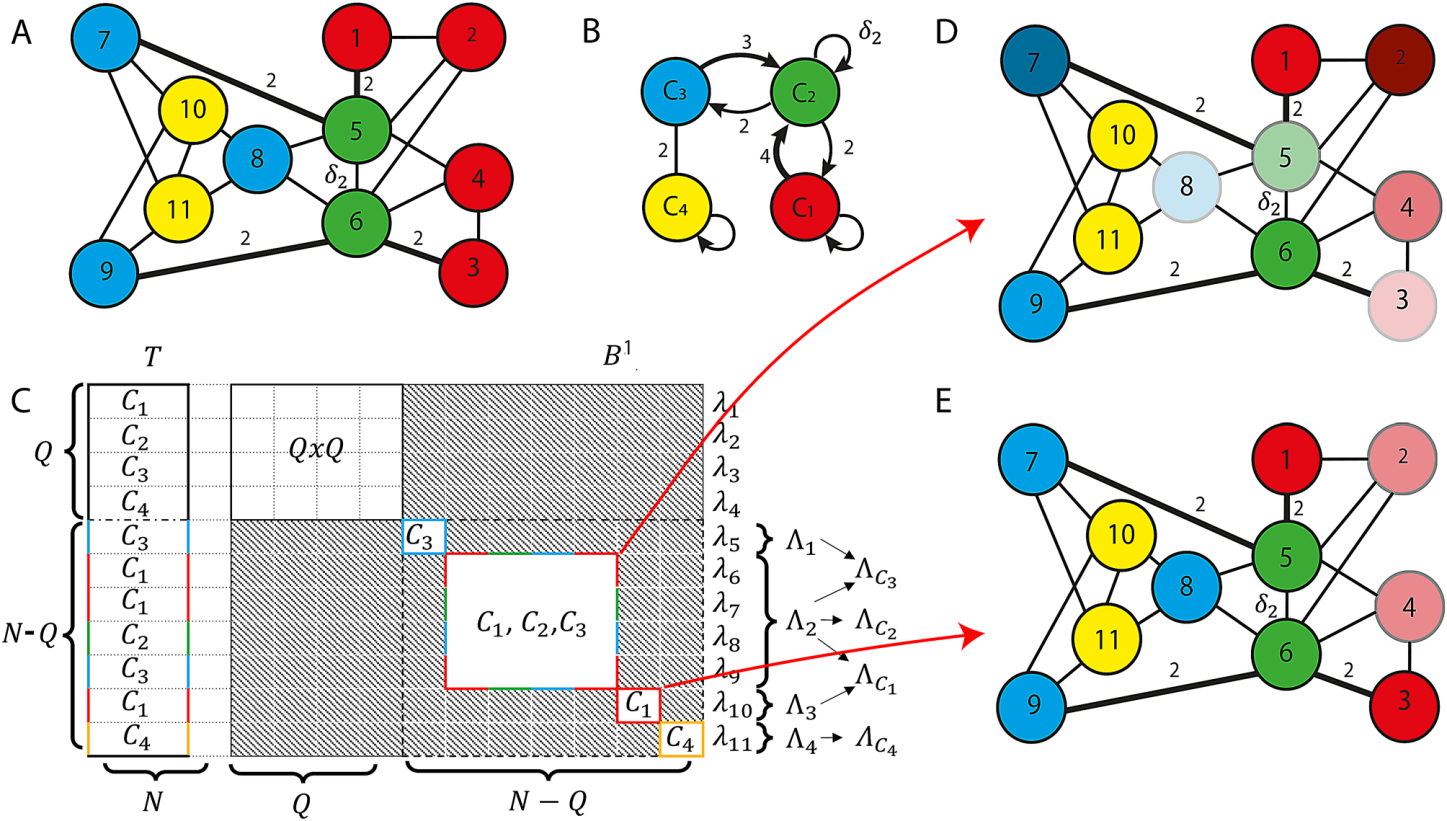
Example. (**A**) Network with $N=11$ nodes, $L=2$ kinds of connection, undelayed ($k=1$) or with delay $\delta _2$ ($k=2$), and $Q=4$ clusters ($C_1 = \{1,2,3,4\},C_2 = \{5,6\},C_3 = \{7,8,9\},C_4 = \{10,11\}$). All connections are bi-directional and with weight 1, with the exception of the thick connections (between nodes 5-7, 1-5, 6-9, 3-6), which have weight 2. The connection between nodes 5 and 6 has the delay $\delta _2$. (**B**) Quotient network corresponding to (**A**). (**C**) Structure of the corresponding matrices *T* and $B^1$, illustrating their relation with the clusters. Network coloring (with a larger number of clusters) after the breaking of the red cluster if its loss of stability is due to the MLEs corresponding to either (**D**) the multi-color sub-block or (**E**) the red sub-block of matrix $B^1$.

Cluster synchronization of the system in Eq. (1) is defined as $x_i(t) = x_j(t)$ for any *t* and for *i*, *j* belonging to the same cluster of a certain partition. The set of the network nodes can be partitioned into equitable clusters (ECs), whose presence is necessary to achieve CS. Indeed, *nodes in the same EC receive the same amount of weighted inputs of a certain type from the other ECs and from the EC itself*. The method we propose for the analysis of CS in networks modeled by Eq. (1) consists of three main steps: (S1) a coloring algorithm to find the *Q* ECs $C_q$ ($q = 1,\ldots ,Q$) of the network, corresponding to a clustering ${\mathcal {C}} = \{C_1,\ldots ,C_Q\}$ (see the example network in Fig. 1A, where $N=11$ and $Q=4$); (S2) a simplified dynamical model (called *quotient network*) whose *Q* nodes correspond to each one of the ECs (see Fig. 1B, which is the quotient network corresponding to Fig. 1A); (S3) an analysis of the cluster stability by linearizing Eq. (1) about a state corresponding to exact synchronization among all the nodes within each cluster.

A detailed description of steps S1 and S2 (with limited or no novelty) is provided in the Supplementary Information. The main novelty of this method is the analysis S3, which is tailored to Eq. (1) following, *mutatis mutandis*, the guidelines defined in previous works for less general networks^15,20^. Step S3 is detailed in Methods. A key step of this analysis is the construction of the matrix *T* that transforms the coupling matrices $A^k$ into block diagonal matrices, $B^k=T A^k T^T$. This corresponds to a change of perturbation coordinates that converts the node coordinate system to the *irreducible representation* (IRR)^15,20,23^ coordinate system, thus evidencing the interdependencies among the perturbation components. For undirected networks, the $N \times N$ matrix *T* can be found as described in^18,20^ (As a technical note for the readers who are familiar with network partitioning, we point out that it was done for the orbital case^20^ and for the equitable single-layer case^18^). For directed networks, the matrix *T* can be constructed (as detailed in Sect. 4 in the Supplementary Information) for two classes of networks: (A) directed networks with clusters containing at most two nodes and (B) directed networks for which directed connections either originate from or end in trivial clusters, i.e., such that $A_{ij}^k \ne A_{ji}^k$ only if either *i* or *j* is in a cluster $C_q$ with $N_q=1$.

The key variational equation that we obtain in all these cases is reported here in compact form for ease of reference:

2 $$\begin{aligned} {\dot{\eta }} = \rho _1 \eta(t) + \rho _2 \eta(t-\delta_k) , \end{aligned}$$

where $\eta = [\eta_{1}^{T}, \eta_{2}^{T},\ldots,\eta_{N}^{T}]^{T}$ and the matrices *ρ*_1_ and *ρ*_2_ are defined in Eq. (5) in the Methods. This equation describes the perturbation dynamics, by separating that along the synchronous manifold (described by the first *Q* components $\eta _i$) from that transverse to it (described by the last components $\eta _i$, $i\in [Q+1, N]$). Through the matrix $\rho _1$ each perturbation $\dot{\eta }_j$ only depends on $\eta _j$, while through the block diagonal matrix $\rho _2$, $\dot{\eta }_j$ also depends on the other perturbation components through the matrices $B^1,\ldots ,B^L$. Therefore, an inspection of the sub-blocks of each matrix $B^k$ allows to quickly check whether there is coupling between the dynamics of perturbations $\eta _i$ and $\eta _j$. To better illustrate this concept, let us consider the undirected, weighted network with $N=11$ nodes, $L=2$ kind of connections, and $Q=4$ clusters ($C_1,C_2,C_3,C_4$) shown in Fig. 1, panel A, with nodes color coded to indicate the ECs they belong to (As a technical note for the readers who are familiar with network partitioning, we point out that the partition of the network nodes is equitable and not orbital^18^). The corresponding quotient network is shown in panel B and is obtained by applying the above definition of EC. For instance, the blue node in panel B corresponds to the EC $C_3$: indeed, each blue node in panel A receives either one connection of type 1 with weight 2 or two connections of type 1 with weight 1 from green nodes and two connections of type 1 with weight 1 from yellow nodes. Notice also the presence of a delay $\delta _2$ in the connection between nodes 5 and 6.

Panel C shows the structure of the matrices *T* (left) and $B^1$ (right) for this network. Notice that matrix $B^2$ has the same structure as $B^1$, whose gray blocks contain only 0 entries. The upper-left $Q\times Q$ block is related to the perturbation dynamics along the synchronous manifold. Each white sub-block in the lower-right $(N-Q)\times (N-Q)$ sub-matrix $B^1_{N-Q}$ (with dashed black borders) describes the perturbation dynamics transverse to the synchronous manifold, thus is associated with loss of synchronization, either transient or permanent depending on the cluster stability. For instance, the $1 \times 1$ yellow (or blue or red) sub-block, is related to cluster $C_4$ (or $C_3$ or $C_1$, respectively), as pointed out in the corresponding row in matrix *T*, and describes the dynamics of the perturbation component $\eta _{11}$ (or $\eta _5$ or $\eta _{10}$, respectively); similarly, the $4 \times 4$ multi-color sub-block corresponds to clusters $C_1,C_2,C_3$. We remark that the structure of this sub-block implies that $\dot{\eta }_6, \dot{\eta }_7, \dot{\eta }_8, \dot{\eta }_9$ depend on $\eta _6, \eta _7, \eta _8, \eta _9$ but not on the other perturbations. Each transverse sub-block has an associated Maximum Laypunov Exponent (MLE) $\Lambda _i$, which can be studied independently from each other.

The stability of each cluster $C_q$ related to one or more sub-blocks depends on the maximum MLE $\Lambda _{C_q}$ among those associated to these sub-blocks: if $\Lambda _{C_q}$ is negative, the cluster $C_q$ is stable, otherwise it is unstable. In the example, we computed the MLE associated to each sub-block: $\Lambda _1$ (blue sub-block), $\Lambda _2$ (multi-color sub-block), $\Lambda _3$ (red sub-block) and $\Lambda _4$ (yellow sub-block). The stability of $C_4$ depends on the sign of $\Lambda _{C_4} = \Lambda _4 = \max \{\lambda _{11}\}$ (i.e., the maximum component of the vector $\lambda _{11}$), whereas the stability of $C_1$ depends on the sign of $\Lambda _{C_1} = \max \{\Lambda _2, \Lambda _3\}$, the stability of $C_2$ depends on the sign of $\Lambda _{C_2} = \Lambda _2$ and the stability of $C_3$ depends on the sign of $\Lambda _{C_3} = \max \{\Lambda _1, \Lambda _2\}$.

Notice that the structure of the matrix $B^1$ allows us to state something more about the cluster stability. Indeed, the red cluster is related to two sub-blocks: the $1 \times 1$ red sub-block and the $4 \times 4$ multi-color sub-block. This means what follows: it is possible for the red cluster to undergo isolated desynchronization (see panel E) if the MLE $\Lambda _3$ becomes positive, while if the MLE $\Lambda _2$ becomes positive, red, blue, and green clusters become unstable together (see panel D). More in general, by inspecting the $B^k_{N-Q}$ block, we can easily determine whether two or more clusters are *intertwined*^15^, namely if the ODEs governing their stability are coupled: if a single sub-block is related to two or more clusters, they are intertwined. This example clearly shows that the stability of each cluster in a subset of intertwined clusters may depend on the stability of the other clusters that belong to the same subset, but is decoupled from the clusters outside of the subset. Therefore, intertwined clusters can lose synchronization without causing a loss of synchronization in the clusters outside the subset, as for the yellow cluster in panel D.

**Figure 2:**
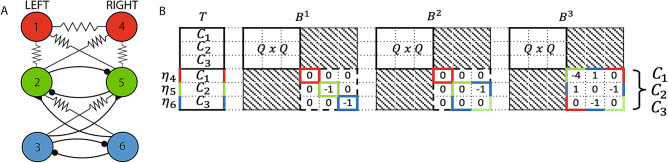
(**A**) Swim CPG of the *Dendronotus iris* nudibranch mollusc. Lines terminating in filled circles indicate inhibitory chemical synapses ($k=1$). Triangles indicate fast excitatory chemical synapses ($k=2$). Resistor symbols indicate electrical (gap junction) connections ($k=3$). Neurons 1-3 are located in the left half of the mollusc brain, neurons 4-6 in the right half of the brain. (**B**) Structure of the matrices *T*, $B^1$, $B^2$, and $B^3$ for the swim CPG network. The gray blocks correspond to 0 entries.

### Case study 1: cluster analysis of the *Dendronotus iris* swim circuit

As a first case study, we apply the proposed method to a *Central Pattern Generator* (CPG), a neural network responsible for organized patterns of organized activities, such as breathing, flying, swimming or walking^24–27^. In particular, we focus on the swim CPG of the *Dendronotus iris* nudibranch mollusc^28–30^. This CPG is composed of six neurons ($N=6$) of the same kind ($M=1$), connected through $L=3$ different kinds of synapses (chemical inhibitory and excitatory, electrical) with no delays, as shown in Fig. 2A. The coupling matrices $A^1$, $A^2$ and $A^3$ are provided in the *dataset S1* of the Supplementary Information.

In this simple network it is quite easy to identify the nodes (belonging to the same EC) that receive the same amount of weighted inputs of a certain type from the other clusters; this directed network has $Q=3$ ECs: $C_1$ (red nodes in Fig. 2A), $C_2$ (green nodes) and $C_3$ (blue nodes). Each cluster contains two nodes, therefore this network belongs to class (A).

Figure 2B shows the structure of the matrices *T* (left) and $B^k$ (right) for the swim CPG network. We remark that the important information is embedded in the matrix structure and not in the values of its non-null entries.

The gray blocks correspond to 0 entries. As usual, in matrices $B^k$, the upper-left $Q\times Q$ block is related to the perturbation dynamics along the synchronous manifold. Each white sub-block in the lower-right $(N-Q)\times (N-Q)$ sub-matrix $B^k_{N-Q}$ describes the perturbation dynamics transverse to the synchronous manifold, thus is associated with loss of synchronization, either transient or permanent depending on the cluster stability.

**Figure 3:**
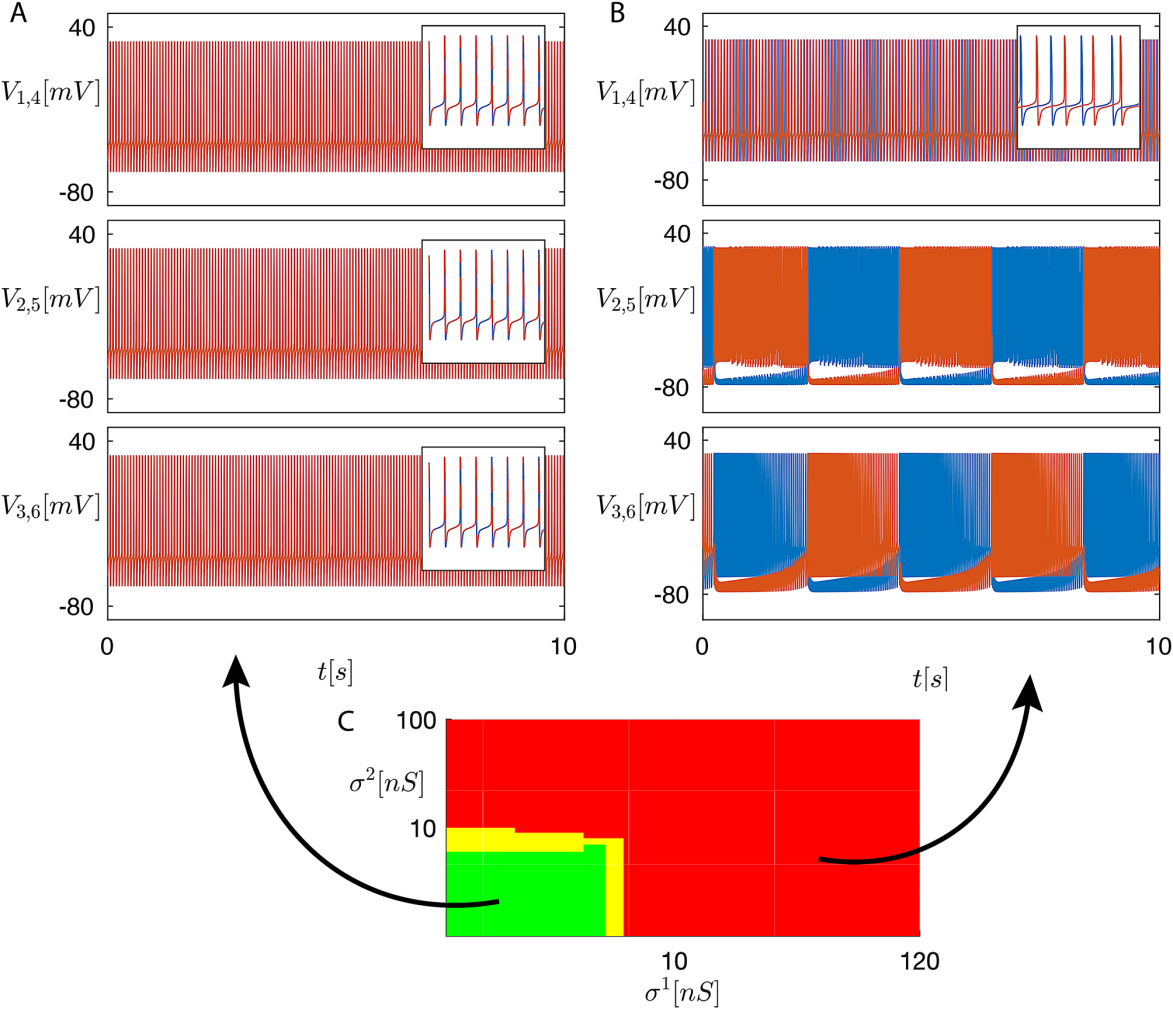
Time plots of the membrane voltages $V_i(t)$ for the swim CPG in normal conditions (**A**) and in conditions emulating (by setting $\sigma ^1 =0$ and $\sigma ^2 = 0$) a bath application of curare (**B**). Cluster $C_1$ (top panel), $C_2$ (middle panel), $C_3$ (bottom panel). Blue lines: $V_i(t)$ for $i = 1, 2, 3$. Red lines: $V_i(t)$ for $i = 4, 5, 6$. (C) Two-parameter map of the stable clusters for the swim CPG. Green region: all clusters ($C_1,C_2,C_3$) are stable (**A**). Red region: $C_1,C_2,C_3$ lose their stability (**B**). Yellow region: bi-stability transition zone.

If we analyze the matrices $B^k$ (related to the *k*-th connection type), we can see that:

- $B^1_{N-Q}$ (related to chemical inhibitory synapses) has three $1 \times 1$ sub-blocks, one per cluster ($C_1$ red, $C_2$ green, $C_3$ blue, according to Fig. 2 in the paper); this implies that for the network with only the chemical inhibitory synapses, the dynamics of the perturbation component $\eta _{4}$ depends only on $\eta _{4}$ through the term $\rho _1$ in Eq. (2), whereas $\dot{\eta }_{5}$ depends only on $\eta _{5}$ through both $\rho _1$ and $\rho _2$ (the same holds for $\dot{\eta }_{6}$, *mutatis mutandis*);
- $B^2_{N-Q}$ (related to chemical excitatory synapses) has one $1 \times 1$ sub-block (with red borders) related to cluster $C_1$ and one $2 \times 2$ sub-block (with dashed green-blue borders) related to clusters $C_2$ and $C_3$; this means that for the network with only the chemical excitatory synapses the dynamics of the perturbation component $\eta _{4}$ depends only on $\eta _{4}$ through the term $\rho _1$ in Eq. (2) (the same holds for $\dot{\eta }_{6}$, *mutatis mutandis*), whereas $\dot{\eta }_{5}$ depends on $\eta _{6}$ through $\rho _2$ and on $\eta _{5}$ through $\rho _1$;
- $B^3_{N-Q}$ (related to electrical synapses) has one $3 \times 3$ sub-block (with dashed multi-color borders) related to all clusters; the structure of this block implies that $\dot{\eta }_4$ depends on $\eta _4$ (through $\rho _1$ and $\rho _2$) and $\eta _5$ (through $\rho _2$), $\dot{\eta }_5$ on $\eta _4$ (through $\rho _2$), $\eta _5$ (through $\rho _1$) and $\eta _6$ (through $\rho _2$), $\dot{\eta }_6$ on $\eta _5, \eta _6$. Therefore, for the network with only the electrical synapses, the clusters $C_1,C_2,C_3$ are intertwined.

In summary, if we consider the whole network, with all kinds of synapses, the three clusters $C_1, C_2, C_3$ are intertwined.

Note that the transverse block is $(N-Q)$-dimensional, so that only intertwined symmetry breakings are possible: this excludes the possibility of isolated loss of synchrony for any of the clusters. In other words, either all the clusters are synchronized or none.

This CPG has been modeled according to previous experimental works^28,30^, using dynamical synapses, as detailed in Methods. This corresponds to state vectors $x_i$ with $n=7$ components. By setting $\sigma ^1 = 120$ nS, $\sigma ^2 = 100$ nS (physiological values^28,30^) and $\sigma ^3 = 0.1$ nS, the CPG oscillates as shown in Fig. 3B.

In cluster $C_1$, the two contralateral neurons emit spikes irregularly, whereas in clusters $C_2$ and $C_3$ the contralateral neurons burst in anti-phase. This means that there are no synchronized clusters in the CPG. This is in perfect agreement with biological measurements^28,30^.

In order to analyze the functional role played by single synapses, neurophysiologists usually use neuroreceptor antagonists (curare in this case^28,30^) to selectively block specific chemical synapses. To simulate this pharmacological effect, we progressively reduced the chemical synaptic strengths $\sigma ^1$ and $\sigma ^2$. The resulting 2D bifurcation diagram, shown in Fig. 3C, is obtained by analyzing the cluster stability on a grid of values of $\sigma ^1$ and $\sigma ^2$, in the ranges [0, 120] nS and [0, 100] nS, respectively. The network exhibits three possible different behaviors, depending on the parameter setting. In the green region, all clusters are stable and the CPG is mono-stable, meaning that it admits only one stable solution, corresponding to these clusters. In particular, the contralateral neurons in clusters $C_2$ and $C_3$ are synchronized, as shown in Fig. 3A, and therefore the CPG does not produce a swimming pattern with left-right alternation. Moreover, the reduction of the synaptic strengths $\sigma ^1$ and $\sigma ^2$ halts bursting activity (In the bursting steady state, the membrane voltage of the neuron is made up of groups of two or more spikes (called bursts) separated by periods of inactivity). Again, this is in excellent agreement with biological measurements^28,30^. In the red region, all clusters become unstable (through a symmetry breaking caused by a subcritical pitchfork bifurcation of cycles), which corresponds to the standard behavior of the swim CPG: in this case, the CPG is again mono-stable and admits only the stable solution shown in Fig. 3B. In the yellow region, the CPG is bi-stable and admits both of the above stable solutions: which one is reached depends on the initial condition. The cluster synchronous solution disappears at the edge between the green and the yellow region, due to a fold of cycle bifurcation of this solution with the unstable solution generated by the symmetry breaking (subcritical pitchfork) bifurcation corresponding to the edge between the yellow and the red region.

As a final remark, we would like to emphasize that “virtually indistinguishable network activity can arise from widely disparate sets of underlying mechanisms, suggesting that there could be considerable animal-to-animal variability in many of the parameters that control network activity, and that many different combinations of synaptic strengths and intrinsic membrane properties can be consistent with appropriate network performance”^31^. This is largely due to the fact that locomotory and other motor functions are controlled through robust mechanisms enabled by homeostatic plasticity and is consistent with the observation of locomotive patterns (even coexisting) that are not generated by exact cluster synchronization^32^. However, by no means this detracts from the potentialities of our analysis method, which considerably expands our ability to understand physiological phenomena and measurements.

### Case study 2: cluster analysis of the macaque cerebral cortex

**Figure 4:**
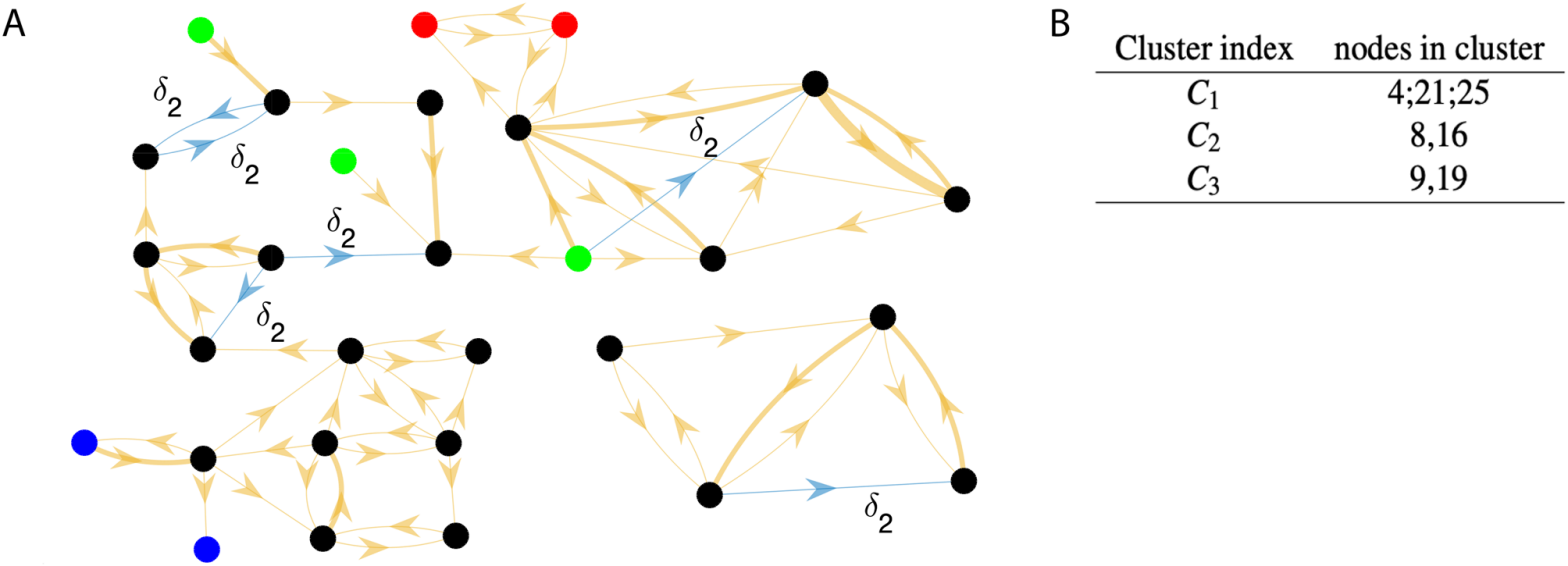
(**A**) Macaque cortical connectivity network: $N = 29$ nodes, $M = 2$ node models, $L=2$ synapse models. Trivial clusters are black. Nodes of the same (non-black) color belong to the same cluster: *C*_1_ (green), *C*_2_ (red), *C*_3_ (blue). (**B**) ECs of the macaque cortical network.

As a second example, following^33–35^, we apply the proposed method to a directed network (shown in Fig. 4) composed of $N = 29$ nodes, each one representing one target area (4 in occipital, 6 in parietal, 6 in temporal, 5 in frontal, 7 in prefrontal, and 1 in limbic regions) among the 91 areas of the macaque cerebral cortex. The neuron models that represent each area are of $M = 2$ kinds: 28 nodes are of kind $i=1$ and one node (corresponding to area V1) is of kind $i=2$, which is due to this one node receiving a visual input^35^. The nodes are connected through $L=2$ kinds of chemical excitatory synapses: one (for $k=1$) that transmits undelayed signals with $\delta _1 = 0$ (in yellow), one (for $k=2$) with delay $\delta _2 > 0$ (in blue).

The overall network is modeled by using the neuron and synapse equations described in Methods and the coupling matrices $A^1$ and $A^2$ provided in the Supplementary Information (dataset S2). The measured connection weights^34^, which range between 0 and 0.7636, have been quantized on four levels (0, 0.1, 0.5, 1) by replacing each original weight with the closest one according to the Euclidean distance. After that, physical connections with length lower than 20 mm have been considered instantaneous (i.e., of kind $k=1$) and the corresponding quantized weights have been stored in the matrix $A^1$, whereas those longer than 20mm have been considered delayed (i.e., of kind $k=2$) and the corresponding quantized weights have been stored in the matrix $A^2$. These quantizations are justified by the fact that exact values for the coupling strengths and the delays reported in the literature are inevitably subject to measurement noise, and by the fact that, as we will see, they lead to the observation of functional mechanisms which are in agreement with physiological data, despite our simplifications.

The network non-trivial equitable clusters (consisting of more than one node) are shown in Fig. 4, where in panel A nodes of the same color (excluding black) belong to the same cluster: green for $C_1$, red for $C_2$ and blue for $C_3$. All nodes in trivial orbits are colored black. Obviously, the presence of a large number of trivial clusters does not mean that the corresponding areas are independent: they are densely connected, as evidenced in Fig. 4A, but they cannot be exactly synchronized.

Despite the rough quantizations applied to synaptic weights and delays, the clusters displayed in Fig. 4B are consistent with some previously reported physiological findings. For instance, cluster $C_2$ contains the nodes corresponding to visual areas 8l and 9/46v in the prefrontal cortex, which are known to be physically close and with similar connections^34,36^. The same holds for cluster $C_3$, which contains the nodes corresponding to the posterior and anterior portion of the inferotemporal cortex (TEO and TEpd, respectively).

**Figure 5:**
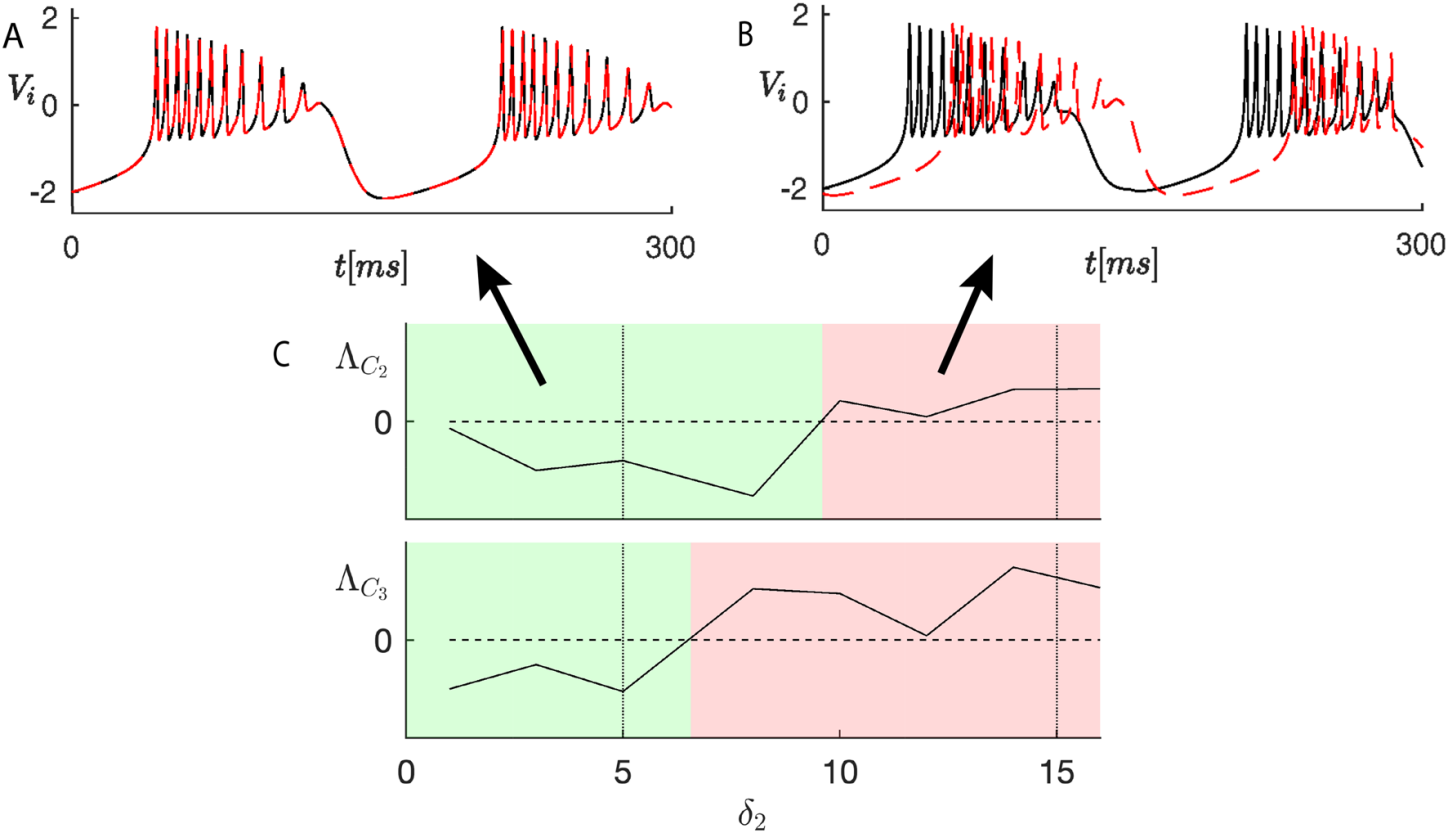
Time plots $V_i(t)$ for different values of $\delta _2$ (5 ms (**A**), 15 ms (**B**)) for cluster $C_3$. (**C**) MLE $\Lambda _{C_q}$ of each cluster $C_q$ ($q=2,3$) vs. coupling delay $\delta _2$, for the macaque cortical connectivity network. Horizontal dashed lines: edge of stability. Vertical dotted lines: $\delta _2$ values corresponding to the time plots in panels A and B.

The directed connections originate from or go to trivial clusters only, therefore this network belongs to class (B), hence its cluster stability can be analyzed through the proposed approach. The structure of the matrices *T* (left) and $B^k$ (right) is provided and commented in the Supplementary Information (Sect. 6), leading to the conclusion that the three clusters $C_1, C_2, C_3$ are not intertwined. The stability analysis has been carried out by varying the delay $\delta _2$ between 0 and 16 ms (8 evenly spaced values). The neurons belonging to cluster $C_1$ do not receive any synaptic inputs, therefore the cluster transverse MLE is $\Lambda _{C_1} = 0$ for any value of $\delta _2$. Figure 5C, shows the MLEs $\Lambda _{C_q}$ of the other clusters $C_q$ ($q=2,3$) versus the delay $\delta _2$. The green (red) regions in each plot $\Lambda _{C_q}(\delta _2)$ denote stability (instability) of the corresponding cluster $C_q$.

The vertical dotted lines mark the $\delta _2$ values corresponding to the time plots shown in the upper panels of Fig. 5: $\delta _2 = 5$ ms (A) and $\delta _2 = 15$ ms (B). These plots display the first state variable $V_i$ of the neurons in cluster $C_3$. The panels show a window of 300 ms after a transient of 19.5 s. The breaking of this cluster is caused by a supercritical pitchfork bifurcation of cycles at each transition between the red and green regions, which generates two smaller stable trivial sub-clusters, each one producing one of the membrane voltages (black or red) in panel B.

**Figure 6:**
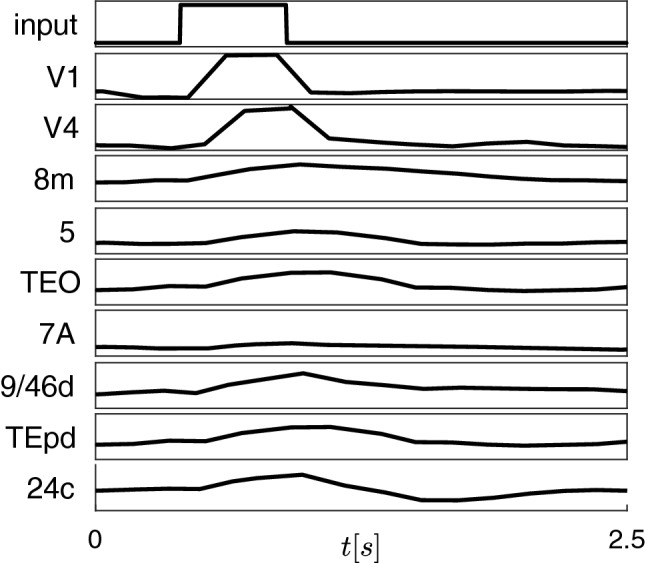
Time responses (firing rates) to a pulse-shaped input to area V1.

From Fig. 5B it clearly emerges that the two neurons in cluster $C_3$ display a phase lag for $\delta _2 = 15$ ms. The synchronization of macaque visual cortex areas in response to visual stimuli has been observed in many experiments^35,37^. In particular, the areas 8l and 9/46v respond in a very similar way to visual inputs to area V1^35^. We thus set $\delta _2=5$ms in order to ensure synchronization of these two areas.

We proceeded to validate our model against the quantizations applied to the synaptic weights and axon delays, described before. To this end, following^35^ we simulated its response to a pulsed input to the primary visual cortex (area V1). The response is propagated up the visual hierarchy, progressively slowing as it proceeds, as shown in Fig. 6. Early visual areas, such as V1 and V4, exhibit fast responses. By contrast, prefrontal areas, such as 8m and 24c, exhibit slower decays to the standard firing rate, with traces of the stimulus persisting several seconds after stimulation. This is in agreement with previous results^35^, which unveil a circuit mechanism for hierarchical processing of visual stimuli in the macaque cortex. Moreover, Fig. 6 evidences CS of the areas TEO and TEpd, corresponding to cluster $C_3$, as predicted by Fig. 5.

As a final remark, we point out that we analyzed the network as in^35^, in order to make fair comparisons. Nonetheless, the four nodes on the bottom right of panel A are disconnected from the rest of the network and are all black, meaning that they all belong to trivial clusters. Therefore, as these nodes cannot form nontrivial clusters, they could have been neglected in the analysis.

## Discussion

The scientific literature counts many papers devoted to the analysis of cluster synchronization. Despite this, a modeling framework that can be applied to study cluster synchronization in neuron networks is still missing. This is due to the peculiar characteristics of this kind of networks, such as heterogeneous neuron populations, characterized by different models or parameters, and heterogeneous directed and undirected synapses, with different communication delays, and whose strength may vary dynamically and nonlinearly based on the state of both pre-synaptic and post-synaptic neurons. The framework proposed in this paper is a fundamental step towards a method that fills this gap by enabling the analysis of cluster synchronization in any network with these features.

Previous works can be seen as particular cases of the proposed framework. For instance, reference^14^ has considered cluster synchronizations by assuming a coupling in the form of Eq. (1) and homogeneous nodal dynamics ($M=1$). Reference^19^ has considered the same problem with the same formalism, but with heterogeneous nodes ($M>1$). In both cases, the analysis is limited to finding the clusters, without analyzing their stability with a variational method. Other papers have studied networks with coupling depending only on either $x_j$^18^ or $x_i - x_j$ (diffusive or Laplacian coupling)^17^, but without consideration of communication delays.

The proposed method has allowed us to study and characterize cluster synchronization in two case studies of interest to the neuroscience community, and to find results in agreement with biological observations. The two examples are relatively simple, in terms of network complexity, but the approach outlined in the paper can be applied to more complex situations with more parameter variations among the individual oscillators (or completely different oscillators) as well as more values of the delays. The availability of a method for the analysis of this kind of networks is key to enabling further studies and to filling the existing gap between modeling and neuroscience. For instance, it is widely accepted that the balance between excitation and inhibition in connected sub-populations of neurons^38,39^ and the network structure (and in particular the presence of neuron modules or clusters) strongly affect the information transmission between neuronal assemblies^40^ and might play significant roles in processes ranging from simple sensory transmission to perception and attention as well as learning and termination of ongoing population activity (see^41^ and references therein). Moreover, studies show that neurophysiological heterogeneity in the cortex has clear influences on functional connectivity^35,42^. Therefore, the proposed method can be used to study cluster synchronization in these networks, as shown in the second case study. In addition, our method could be used as a diagnostic tool to distinguish between pathological and non-pathological situations characterized by different patterns of cluster synchronization^16^ and as a simulation tool to perform virtual experiments and to reduce the number of actual experiments.

What are the limits of the proposed approach? A first limitation lies in the class of networks that can be completely analyzed. Many neuron networks contain both recurrent and feedforward connections, they are directed and do not belong to the two classes (A) and (B) that allow for an analysis of the interdependencies among synchronized clusters. Extending the proposed approach to a wider class of networks will be the subject of future research.

A second limitation is that, in the presence of delays $\delta _k$, the network can admit other synchronous solutions^43^, which cannot be predicted by our method. In particular, when the coupled dynamics is periodic, it is possible that signals that propagate with different transmission delays become indistinguishable from each other^44,45^. For example, a delay that is equal to the oscillations period would generate a signal that is identical to the one in which no delay is present: as a result, connections that are treated with our method as different, are indeed identical. On the other hand, when the oscillatory behavior is very regular, it is also possible that time delay can cause two interactions to cancel with each other^46^, thus resulting in a change of the effective network topology.

As a third limitation, we point out that the proposed model is completely deterministic and assumes that a reliable model of the network is available. These are quite strong modeling assumptions, since in real neuron networks the presence of noise is unavoidable and not always neuron and synapse models can be determined accurately. Despite this and despite the absence of information about the basins of attraction of stable clusters, our approach can provide useful information. As stated in the Introduction, in a real network cluster synchronization will be approximate^47^, not exact, as measured by high correlation values between the membrane potentials of the neurons/nodes belonging to a given stable cluster. In this perspective, the patterns found with the proposed method are approximations to some more realistic solutions, which are characterized by higher complexity. Our analysis method is far from providing an accurate description of the dynamics of real neuron networks. This notwithstanding, it can help understanding basic cluster synchronization mechanisms, whose robustness can be checked by resorting to other less deterministic approaches. To this end, as stated in the introduction, we resort to the *Occam’s razor principle* and focus on deterministic models, but remove the assumption of identical dynamics and extend the applicability of tools for the identification and analysis of cluster synchronization^20^. In other words, in order to apply our method, we *need* to simplify in some reasonable way the real network (as done through quantization of some parameters in the case study 2) for finding exact clusters and the exact clusters that we find are approximations of the real (intrinsecally imperfect) clusters.

As a final remark, in this paper we focused on neuron networks, modeling them as multi-layer networks, where each layer corresponds to a different kind of neuron (thus leading to an *M*-layer network) and we can have both intra-layer and inter-layer connections. The proposed approach can be applied to other neuron-like multi-layer networks of oscillators, provided that they can be described through the proposed formalism. For instance, cluster synchronization in arrays of spin-torque oscillators^48^ or semiconductor laser arrays^49^ could be analyzed through the proposed method.

## Methods

### Step S3: analyzing cluster stability

Here we present the method to analyze stability of clusters for the case of both nodes and connections of different types and for coupling functions that depend not only on the state ($x_j$) of the nodes directly connected to the *i*-th cell, but also on the cell’s own state $x_i$. In a previous work^20^, two of the authors proposed a similar analysis for the simpler case (not related to neurons) in which there are no communication delays and no dependence of the coupling function on $x_i$. The approach grounds on two main steps: (i) writing the variational equations of the network about the synchronized solutions and (ii) expressing these variational equations in a new system of coordinates, which decouples the perturbation dynamics along the transverse manifold from that along the synchronous manifold.

We collect all state trajectories in the vector ${x}(t) = [x_1^T(t), x_2^T(t),\dots ,x_N^T(t)]^T$. As it is possible for all the nodes within a cluster to synchronize, we define the *q*-th cluster state: $s_q(t) = x_i(t)$ for all *i* in cluster $C_q$. Correspondingly, the network can produce *Q* distinct synchronized motions $\{s_1(t), s_2(t),\ldots ,s_Q(t)\}$, one per cluster. We collect them in the vector ${s}(t) = [s_1^T(t), s_2^T(t),\dots ,s_Q^T(t)]^T$.

We analyze the dynamics of a small perturbation $w_i(t) = x_i(t)-s_{q_i}(t)$ ($i=1,\ldots ,N$), where $s_{q_i}(t)$ is the *q* cluster state for node *i* in cluster $C_q$, by linearizing around a specific network solution ${s}(t)$,

3 $$\begin{aligned} {\dot{w}}_i(t)&= \displaystyle {D{\tilde{f}}_i(s_{q_i}(t)) w_i(t) + \sum _{k=1}^{L}\sigma ^k\sum _{j=1}^N A^k_{ij} D_1 h^k (s_{q_i}(t),s_{q_j}(t-\delta _k)) w_i(t)} \nonumber \\&\quad {+ \sum _{k=1}^{L}\sigma ^k\sum _{j=1}^N A^k_{ij} D_2 h^k (s_{q_i}(t),s_{q_j}(t-\delta _k)) w_j(t-\delta _k)}, \end{aligned}$$

where $D_i$ is the Jacobian operator computed with respect to the *i*-th argument of the function at which it is applied (subscript omitted if the function has only one argument).

All perturbations are collected in a column vector $w(t) =[w_1^T(t), \ldots ,w_N^T(t)]^T$ of length *Nn*, with $w_i \in {\mathbb {R}}^n$. Note that, due to the assumption of cluster synchronization, nodes within the generic cluster $C_q$ share the same state ($x_i(t)=x_j(t) = s_q(t),\ \forall t \Leftrightarrow i,j\in C_q$) and their isolated dynamics is described by the same function $f_q$. Hence, it is possible to describe the perturbation dynamics as in Eq. (4),

4 $$\begin{aligned} \dot{w}(t)= & {} \displaystyle {\left[ \sum _{q=1}^Q E_{C_q} \otimes \left( Df_q(s_q(t))+\sum _{k=1}^{L}\sigma ^k \sum _{p=1}^Q R^k_{qp} D_1 h^k(s_q(t),s_p(t-\delta _k))\right) \right] } {w}(t)\nonumber \\&+ \displaystyle {\sum _{k=1}^{L} \left[ \sigma ^k \sum _{q=1}^Q \sum _{p=1}^P \left( (E_{C_q} A^k E_{C_p}) \otimes D_2 h^k(s_q(t),s_p(t-\delta _k))\right) \right] w(t-\delta _k)} \end{aligned}$$

where $R^k$ is the weighted adjacency matrix for the quotient network and for the connections of kind *k* (obtained as detailed in the Supplementary Information (Sect. 3)), $\otimes$ is the Kronecker product operator and the $N \times N$ diagonal matrix $E_{C_q}$ has entries $E_{C_q,ii}=1$, if node $i \in C_q$, 0 otherwise, i.e., this matrix identifies all the nodes *i*’s belonging to cluster $C_q$.

Notice that the presence of different neuron models determines different expressions for the Jacobian matrices $D f_q$ in Eq. (4). Equation (4) is quite general: it is valid for both directed and undirected networks. What follows, instead, holds for undirected networks and for two classes of directed networks: (A) directed networks with clusters containing at most two nodes and (B) directed networks for which directed connections either originate from or end in trivial clusters, i.e., such that $A_{ij}^k \ne A_{ji}^k$ only if either *i* or *j* is in a cluster $C_q$ with $N_q=1$. In these cases, we are able to find the *irreducible representations* of the multi-layer network symmetry group^15,20,23^, that is a change of coordinates $\eta =(T \otimes {\mathbb {I}}_{n}) w$ that converts the node coordinate system to the IRR coordinate system, thus evidencing the interdependencies among the perturbation components. This change of coordinates requires attention, as for the case of Eq. (1) the interaction term $h^k$ depends not only on $x_j$ but also on $x_i$, contrary to what was assumed in previous works^15–18,20,50^.

For undirected networks, the $N \times N$ matrix *T* can be found as described in previous works^18,20^. For directed networks of kind (A) and (B), the matrix *T* can be constructed as described in the Supplementary Information (Sect. 4). By applying the transformation *T* to Eq. (4), we obtain Eq. (5),

5 $$\begin{aligned} {\dot{\eta }}(t)= & {} \displaystyle {\left[ \underbrace{\sum _{q=1}^Q J_{q} \otimes \left( Df_q(s_q(t))+\sum _{k=1}^{L}\sigma ^k \sum _{p=1}^Q R^k_{qp} D_1 h^k(s_q,s_p(t-\delta _k))\right) }_{\rho _1} \right] } \eta (t)\nonumber \\&+ \displaystyle {\underbrace{\sum _{k=1}^{L} \sigma ^k \sum _{q=1}^Q\sum _{p=1}^Q \left( (J_{q} B^k J_{p}) \otimes D_2 h^k(s_q(t),s_p(t-\delta _k))\right) }_{\rho _2} \eta }(t-\delta _k) \end{aligned}$$

where $J_{q}=TE_{C_q}T^T$ and $B^k=T A^k T^T$. Notice that the change of coordinate is orthonormal, so that $T^T=T^{-1}$. As proved in the Supplementary Information (Sect. 5), $J_q$ is diagonal.

For undirected networks, each matrix $B^k$ (and therefore also $J_q B^k J_p$) is block diagonal with two blocks: the upper-left of size $Q\times Q$ and the lower-right ($B^k_{N-Q}$) of size $(N-Q)\times (N-Q)$. Therefore, through the IRR change of coordinates we have decoupled the perturbation dynamics along the synchronous manifold (described by the first *Q* components $\eta _i$) from that transverse to it (described by the last components $\eta _i$, $i\in [Q+1, N]$). Moreover, each matrix $B^k_{N-Q}$ is in turn block diagonal: as a consequence, the behavior of a perturbation with respect to the synchronous solution can be studied by considering many independent, smaller-size problems, each one related to one or more clusters^18^. In this way the stability of the synchronized clusters can be calculated using the separate, simpler, lower-dimensional ODEs of the transverse sub-blocks. We remark that $\dot{\eta }_j$ depends on $\eta _i$ only through the matrix $J_q B^k J_p$, as $J_q$ is diagonal (see Eq. (5)). In other words, the term $\rho _1$ in Eq. (5) is a diagonal matrix, which relates $\dot{\eta }_j$ only to $\eta _j$. By contrast, $\rho _2$ relates $\dot{\eta }_j$ also to the other perturbation components. Therefore, an inspection of the sub-blocks of $B^k$ allows to quickly check whether there is coupling between the dynamics of perturbations $\eta _i$ and $\eta _j$. Since the stability of each cluster depends on the evolution of some specific perturbations, the structure of blocks $B^k_{N-Q}$ determines also whether two clusters are intertwined or not.

For directed networks, instead, $J_q B^k J_p$ is in general block upper-triangular with the upper part of size $Q \times N$ and the other of size $(N-Q)\times (N-Q)$. The perturbation dynamics on the synchronous manifold depends in general on all perturbations (synchronous and transverse), whereas on the transverse manifold the perturbation dynamics depends on the transverse perturbations only.

In summary, for all kinds of networks (undirected and directed) we can study the stability of the cluster synchronous solution by computing the Lyapunov exponents corresponding to each transverse perturbation component. Moreover, for undirected networks and for directed networks of kind (A) or (B), we can also find the change of coordinates that provides the minimum-size blocks in the block $B^k_{N-Q}$ of matrix $B^k$. This allows one to detect interdependencies in the stability of different clusters through the MLEs $\Lambda _m$ associated to each sub-block.

We can study the stability of clusters in terms of the Lyapunov exponents $\lambda _{i_j}$ (with $i = 1,\ldots ,N$ and $j = 1,\ldots ,n$), collected in vectors $\lambda _i \in {\mathbb {R}}^n$ and corresponding to the generic perturbation $\eta _i(t) \in {\mathbb {R}}^n$.

In general, the first *Q* vectors $\lambda _i$ correspond to the perturbation along the synchronous manifold, thus they are not related to the cluster stability; we are interested only in determining the vectors of Lyapunov exponents corresponding to the perturbations transverse to the synchronous manifold, namely $\lambda _{Q+1},\ldots \lambda _N$. Therefore, each sub-block of $J_q B^k J_p$ is related to a subset of Lyapunov vectors $\lambda _i$, as shown in Fig. 1C, rightmost labels. Let *i*(*m*) be the set of indices corresponding to the *m*-th sub-block, i.e., the index of the rows corresponding to the *m*-th sub-block in matrix $J_q B^k J_p$. For instance, in Fig. 1C, $i(1) = 5$ and $i(2) = \{6,7,8,9\}$. Let

6 $$\begin{aligned} \Lambda _m = \max _{\small {\begin{array}{c} i \in i(m)\\ j \in \{1,\ldots ,n\} \end{array}}} \{ \lambda _{i_j}\} \end{aligned}$$

be the MLE related to the *m*-th sub-block. As the perturbations related to each sub-block are independent of those related to other blocks, we can compute the MLEs as follows:

7 $$\begin{aligned} \Lambda _m = \max _{i \in i(m)} \lim _{\Vert \eta _i(0)\Vert \rightarrow 0} \; \lim _{t \rightarrow \infty } \frac{1}{t}\ln {\left( \frac{\Vert \eta _i(t)\Vert _2}{\Vert \eta _i(0)\Vert _2}\right) } \end{aligned}$$

The stability of each cluster $C_q$ related to one or more sub-blocks depends on the MLE $\Lambda _{C_q}$ among those associated to these sub-blocks: if $\Lambda _{C_q}$ is negative, the cluster $C_q$ is stable, otherwise it is unstable.

From a numerical standpoint, since we are finally interested only in the sign of the MLE, the integration of the *i*-th component of the variational equation (5) starts from a random initial condition and is stopped when $\Vert \eta _i(t)\Vert _2$ either overcomes a given threshold ${\bar{\varepsilon }}$ (meaning that the perturbation is diverging) or falls below another threshold ${\underline{\varepsilon }}$, meaning that the perturbation is converging to zero. In the presented results, we set ${{\bar{\varepsilon }}} = 10^4$ and ${\underline{\varepsilon }} = 10^{-4}$.

#### Remark

If the network nodes have different state dimensions $n_i$, the components in excess (used to have the same state length $n= \max _i n_i$) correspond to null Lyapunov exponents, which must be neglected in the stability analysis.

### Models used for the analysis of the swim CPG

Chemical synapses are dynamical and modeled as follows^30^:

8 $$\begin{aligned} {\dot{s}}_{k,j} = \frac{1}{\tau _s} \frac{s_\infty (V_j) - s_{k,j}}{1-s_\infty (V_j)} \end{aligned}$$

where the index *k* denotes inhibitory chemical synapses (for $k=1$), excitatory chemical synapses ($k=2$) and instantaneous electrical synapses ($k=3$), *j* is the index of the pre-synaptic neuron and

$$\begin{aligned} s_\infty (V_j) = {\left\{ \begin{array}{ll} \tanh {\left( \frac{V_j-V_T}{V_s} \right) } &\quad \text {if } V_j > V_T\\ 0 &\quad \text {otherwise}\\ \end{array}\right. } \end{aligned}$$

with $\tau _s = 40$ms, $V_T = -30$mV and $V_s = 25$mV. Notice that each chemical synapse which starts from node *j* has state $s_{k,j}$, which is included into the *j*-th node state vector $x_j$.

The activation functions for dynamical chemical synapses (inhibitory for $k=1$ and excitatory for $k=2$) and instantaneous electrical synapses ($k=3$) are

9 $$\begin{aligned} a^1(V_i,x_j) = (E^1 - V_i)s_{1,j} \quad \quad a^2(V_i,x_j) = (E^2 - V_i)s_{2,j} \quad \quad a^3(V_i,V_j) = V_j-V_i , \end{aligned}$$

with $E^1 = -80$mV, $E^2 = 0$mV.

The neuron model^51^ has 5 state variables, namely $[V_i,h_i,n_i,\chi _i,Ca_i]^T$. Therefore, the state vector $x_i$ has $n=7$ components $[V_i,h_i,n_i,\chi _i,Ca_i,s_{1,i},s_{2,i}]^T$:

10 $$\begin{aligned} {\dot{x}}_i = {\tilde{f}}_i(x_i) = \begin{bmatrix} (-I_{Na} -I_K -I_{Ca} -I_{KCa} -I_{l})/C\\ (h_\infty (V_i) - h_i)/\tau _h(V_i)\\ (n_\infty (V_i) - n_i)/\tau _n(V_i)\\ (\chi _\infty (V_i) - \chi _i)/\tau _\chi (V_i)\\ \rho [K_c \chi _i (V_{Ca} - V_i) - Ca_i]\\ \frac{1}{\tau _s} \frac{s_\infty (V_i) - s_{1,i}}{1-s_\infty (V_i)}\\ \frac{1}{\tau _s} \frac{s_\infty (V_i) - s_{2,i}}{1-s_\infty (V_i)} \end{bmatrix}, \end{aligned}$$

where $C = 1 \mu \text {F}/\text {cm}^2$, $\rho = 0.0001 \text {mV}^{-1}$, $K_c = 0.0085\text {mV}^{-1}$ and $V_{Ca} = -180$mV. Sodium current $I_{Na}$ can be computed as $I_{Na} = g_{Na} m_\infty ^3 h (V_i - V_{Na})$, where $V_{Na} = 30$ mV and $g_{Na} = 4$nS. The fast potassium current $I_K$ is $I_K =g_K n_i^4 (V_i - V_K)$, where the reversal potential is $V_K = -75$mV and the maximum $\hbox {K}^+$ conductance value is $g_K = 0.3$nS. TTX-resistant calcium current $I_{Ca}$: $I_{Ca} = g_{Ca} \chi _i (V_i - V_{Ca})$, where the reversal potential is $V_{Ca} = 140$mV and the maximum $\hbox {Ca}^{2+}$ conductance is $g_{Ca} = 0.03$nS. Outward $\hbox {Ca}^{2+}$-activated $\hbox {K}^+$ current: $I_{KCa} =g_{KCa} \frac{Ca_i}{0.5+Ca_i} (V_i-V_K)$, where the reversal potential is $V_{K} = -75$mV. Leak current $I_{l}$: $I_{l} = g_L(V_i - V_L)$, where the reversal potential $V_L = -40$mV and the maximum conductance value is $g_L = 0.0003$nS. $m_\infty$ is defined as $m_\infty = \frac{\alpha _m}{\alpha _m + \beta _m}$, where $\alpha _m = 0.1 \frac{50-V_s}{e^{(50-V_s)/10}-1}$ and $\beta _m =4 e^{((25-V_s )/18)}$, with $V_s = \frac{127V_i + 8265}{105}$.

Auxiliary functions for $h_i$:

$$\begin{aligned} h_\infty = \frac{\alpha _h}{\alpha _h + \beta _h} \quad \text {and} \quad \tau _h = \frac{12.5}{\alpha _h + \beta _h}, \end{aligned}$$

where $$\alpha _h = 0.07 e^{((25-V_s )/20)}$$ and $$\beta _h = \frac{1}{e^{(55-V_s)/10}+1}$$.

Auxiliary functions for $n_i$:

$$\begin{aligned} n_\infty = \frac{\alpha _n}{\alpha _n + \beta _n} \quad \text {and} \quad \tau _n = \frac{12.5}{\alpha _n + \beta _n}, \end{aligned}$$

where $$\alpha _n = \frac{55-V_s}{e^{(55-V_s)/10}-1}$$ and $$\beta _n = 0.125 e^{((45-V_s )/80)}$$.

Auxiliary functions for $\chi _i$:

$$\begin{aligned} \chi _\infty = \frac{1}{1 + e^{-0.3(V_i-40)}} \quad \text {and} \quad \tau _\chi = 9400 \text {ms}. \end{aligned}$$

### Models used for the analysis of the macaque cortical network

Each node of the network has been modeled through the Hindmarsh-Rose neuron model^52^:

11 $$\begin{aligned} {\dot{x}}_i = {\tilde{f}}_i(\underbrace{x_i}_{[V_i,y_i,z_i]^T}) = \begin{bmatrix} y_i - V_i^3 + bV_i^2 - z_i + I_i\\ 1-5V_i^2 - y_i\\ \mu (s (V_i-x_{rest}) - z_i) \end{bmatrix}, \end{aligned}$$

with $b=2.7$, $\mu = 0.01$, $s=4$, $x_{rest} = -1.6$, and $I_1=2$ or $I_2=3$, which distinguish the two node models.

The excitatory synapse activation functions $a^k$ ($k=1,2$) are defined according to the fast threshold modulation paradigm^53^:

12 $$\begin{aligned} a^k(V_i,V_j) = \frac{E - V_i}{1+e^{\nu (V_j-\theta )}}, \end{aligned}$$

with $E = 2$, $\nu =10$ and $\theta = -0.6$. Therefore all synapses are instantaneous, but the membrane potentials transmitted through electrical synapses are not delayed ($\delta _1 = 0$), whereas those transmitted through chemical synapses are delayed ($\delta _2 \ne 0$).

## Supplementary information

Supplementary Information.

## References

[1] Bassett DS, Zurn P, Gold JI (2018). On the nature and use of models in network neuroscience. Nat. Rev. Neurosci..

[2] Herz AV, Gollisch T, Machens CK, Jaeger D (2006). Modeling single-neuron dynamics and computations: a balance of detail and abstraction. Science.

[3] Kreiter AK, Singer W (1996). Stimulus-dependent synchronization of neuronal responses in the visual cortex of the awake macaque monkey. J. Neurosci..

[4] Maldonado PE, Friedman-Hill S, Gray CM (2000). Dynamics of striate cortical activity in the alert macaque: II. Fast time scale synchronization. Cereb. Cortex.

[5] Glennon M, Keane MA, Elliott MA, Sauseng P (2016). Distributed cortical phase synchronization in the EEG reveals parallel attention and working memory processes involved in the attentional blink. Cereb. Cortex.

[6] Bullmore E, Sporns O (2009). Complex brain networks: graph theoretical analysis of structural and functional systems. Nat. Rev. Neurosci..

[7] Guevara Erra R, Perez Velazquez JL, Rosenblum M (2017). Neural synchronization from the perspective of non-linear dynamics. Front. Comput. Neurosci..

[8] Winfree AT (2001). The Geometry of Biological Time.

[9] Nakao H, Yanagita T, Kawamura Y (2014). Phase-reduction approach to synchronization of spatiotemporal rhythms in reaction-diffusion systems. Phys. Rev. X.

[10] Brown E, Moehlis J, Holmes P (2004). On the phase reduction and response dynamics of neural oscillator populations. Neural Comput..

[11] Tikidji-Hamburyan RA, Leonik CA, Canavier CC (2019). Phase response theory explains cluster formation in sparsely but strongly connected inhibitory neural networks and effects of jitter due to sparse connectivity. J. Neurophysiol..

[12] Seress Á (2003). Permutation Group Algorithms.

[13] Stein W, Joyner D (2005). SAGE: system for algebra and geometry experimentation. ACM Sigsam Bull..

[14] Belykh I, Hasler M (2011). Mesoscale and clusters of synchrony in networks of bursting neurons. Chaos.

[15] Pecora LM, Sorrentino F, Hagerstrom AM, Murphy TE, Roy R (2014). Cluster synchronization and isolated desynchronization in complex networks with symmetries. Nat. Commun..

[16] Sorrentino F, Pecora LM, Hagerstrom AM, Murphy TE, Roy R (2016). Complete characterization of the stability of cluster synchronization in complex dynamical networks. Sci. Adv..

[17] Cho YS, Nishikawa T, Motter AE (2017). Stable chimeras and independently synchronizable clusters. Phys. Rev. Lett..

[18] Siddique AB, Pecora L, Hart JD, Sorrentino F (2018). Symmetry-and input-cluster synchronization in networks. Phys. Rev. E.

[19] Lodi, M., Della Rossa, F., Sorrentino, F. & Storace, M. An algorithm for finding equitable clusters in multi-layer networks. In *2020 IEEE International Symposium on Circuits and Systems (ISCAS)* 1–4 (IEEE, 2020).

[20] Della Rossa F (2020). Symmetries and cluster synchronization in multilayer networks. Nat. Commun..

[21] Boccaletti S (2014). The structure and dynamics of multilayer networks. Phys. Rep..

[22] Mattia M, Biggio M, Galluzzi A, Storace M (2019). Dimensional reduction in networks of non-markovian spiking neurons: equivalence of synaptic filtering and heterogeneous propagation delays. PLoS Comput. Biol..

[23] Golubitsky M, Stewart I, Schaeffer DG (2012). Singularities and Groups in Bifurcation Theory.

[24] Grillner S (2006). Biological pattern generation: the cellular and computational logic of networks in motion. Neuron.

[25] Ijspeert AJ (2008). Central pattern generators for locomotion control in animals and robots: a review. Neural Netw..

[26] Goulding M (2009). Circuits controlling vertebrate locomotion: moving in a new direction. Nat. Rev. Neurosci..

[27] Kiehn O, Dougherty K, Pfaff D, Volkow N (2016). Locomotion: circuits and physiology. Neuroscience in the 21st Century: From Basic to Clinical.

[28] Sakurai A, Newcomb JM, Lillvis JL, Katz PS (2011). Different roles for homologous interneurons in species exhibiting similar rhythmic behaviors. Curr. Biol..

[29] Newcomb JM, Sakurai A, Lillvis JL, Gunaratne CA, Katz PS (2012). Homology and homoplasy of swimming behaviors and neural circuits in the nudipleura (mollusca, gastropoda, opisthobranchia). Proc. Natl. Acad. Sci. USA.

[30] Sakurai A, Katz PS (2017). Artificial synaptic rewiring demonstrates that distinct neural circuit configurations underlie homologous behaviors. Curr. Biol..

[31] Prinz AA, Bucher D, Marder E (2004). Similar network activity from disparate circuit parameters. Nat. Neurosci..

[32] Canavier CC (1997). Phase response characteristics of model neurons determine which patterns are expressed in a ring circuit model of gait generation. Biol. Cybern..

[33] Markov NT (2013). Cortical high-density counterstream architectures. Science.

[34] Markov NT (2014). A weighted and directed interareal connectivity matrix for macaque cerebral cortex. Cereb. Cortex.

[35] Chaudhuri R, Knoblauch K, Gariel M-A, Kennedy H, Wang X-J (2015). A large-scale circuit mechanism for hierarchical dynamical processing in the primate cortex. Neuron.

[36] Goulas A, Schaefer A, Margulies DS (2015). The strength of weak connections in the macaque cortico-cortical network. Brain Struct. Funct..

[37] Bosman CA (2012). Attentional stimulus selection through selective synchronization between monkey visual areas. Neuron.

[38] Vogels TP, Abbott L (2009). Gating multiple signals through detailed balance of excitation and inhibition in spiking networks. Nat. Neurosci..

[39] Isaacson JS, Scanziani M (2011). How inhibition shapes cortical activity. Neuron.

[40] Shein-Idelson M, Cohen G, Ben-Jacob E, Hanein Y (2016). Modularity induced gating and delays in neuronal networks. PLoS Comput. Biol..

[41] Uzuntarla M, Torres JJ, Calim A, Barreto E (2019). Synchronization-induced spike termination in networks of bistable neurons. Neural Netw..

[42] Turk E, Scholtens LH, van den Heuvel MP (2016). Cortical chemoarchitecture shapes macroscale effective functional connectivity patterns in macaque cerebral cortex. Hum. Brain Mapp..

[43] Golubitsky M, Stewart I (2006). Nonlinear dynamics of networks: the groupoid formalism. Bull. Am. Math. Soc..

[44] Choe CU, Dahms T, Hövel P, Schöll E (2010). Controlling synchrony by delay coupling in networks: from in-phase to splay and cluster states. Phys. Rev. E.

[45] Williams CR, Sorrentino F, Murphy TE, Roy R (2013). Synchronization states and multistability in a ring of periodic oscillators: Experimentally variable coupling delays. Chaos.

[46] Zakharova A (2013). Time delay control of symmetry-breaking primary and secondary oscillation death. EPL.

[47] Sorrentino F, Pecora L (2016). Approximate cluster synchronization in networks with symmetries and parameter mismatches. Chaos.

[48] Zaks M, Pikovsky A (2017). Chimeras and complex cluster states in arrays of spin-torque oscillators. Sci. Rep..

[49] Shena J, Hizanidis J, Kovanis V, Tsironis GP (2017). Turbulent chimeras in large semiconductor laser arrays. Sci. Rep..

[50] Schaub MT (2016). Graph partitions and cluster synchronization in networks of oscillators. Chaos.

[51] Plant RE (1981). Bifurcation and resonance in a model for bursting nerve cells. J. Math. Biol..

[52] Hindmarsh JL, Rose R (1984). A model of neuronal bursting using three coupled first order differential equations. Proc. R. Soc. Lond. B.

[53] Somers D, Kopell N (1993). Rapid synchronization through fast threshold modulation. Biol. Cybern..

