# Supplementary material for: Analyzing synchronized clusters in neuron networks
Source: Sci Rep. 2020 Oct 1;10:16336. doi: 10.1038/s41598-020-73269-9 (PMC7530773; doi:10.1038/s41598-020-73269-9)
Supplement: Supplementary file 1 — Supplementary Information. [file 41598_2020_73269_MOESM1_ESM.pdf]

Supplemental Material for Analyzing synchronized clusters in neuron networks

Matteo Lodi,[1] Fabio Della Rossa,[2,3] Francesco Sorrentino,[2] Marco Storace [1*]

[1]DITEN, University of Genoa, Via Opera Pia 11a, I-16145, Genova, Italy
[2]Mechanical Engineering Department, University of New Mexico, Albuquerque, NM 87131, USA
[3]Dipartimento di Elettronica, Informazione e Bioingegneria, Politecnico di Milano, I-20133 Milan, Italy

*To whom correspondence should be addressed; E-mail: marco.storace@unige.it.

August 31, 2020

1 Example 1

To illustrate the proposed formalism, we consider the simple network (composed of $N = 3$ nodes of $M = 2$ kinds of neurons and $L = 1$ kind of synapse) shown in Fig. 1.

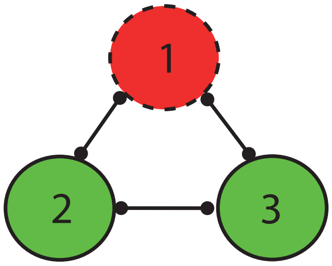

Figure 1: Example network with $N = 3$ nodes of $M = 2$ kinds of neurons (dashed red circle and solid green circle).

Node/neuron 1 is described by the Hindmarsh-Rose model [1]:

$$\begin{cases} \dot{V}_1 = y_1 - V_1^3 + bV_1^2 - z_1 + I_1 \\ \dot{y}_1 = 1 - 5V_1^2 - y_1 \\ \dot{z}_1 = \mu(s(V_1 - x_{rest}) - z_1) \end{cases},$$

whereas nodes 2 and 3 are described by the FitzHugh-Nagumo model [2]:

$$\begin{cases} \dot{V}_i = V_i - \frac{1}{3}V_i^3 + y_i + I_i \\ \dot{y}_i = a + bV_i - cy_i \end{cases} \qquad i = 2, 3.$$

Nodes are connected according to the following adjacency matrix:

$$A = \begin{bmatrix} 0 & 1 & 1 \\ 1 & 0 & 1 \\ 1 & 1 & 0 \end{bmatrix}$$

The synapses are described by the so-called dynamical α-synapse model [3]. The state of each synapse originating from the j-th neuron is s_j^1 and evolves according to the following model:

$$\dot{s}_j^1 = \alpha(1 - s_j^1)\frac{1}{1 + e^{\nu(V_j - V_{th})}} - \beta s_j^1.$$

The corresponding activation a^1 depends on the post-synaptic membrane potential V_i: $a^1(V_i(t), x_j(t - \delta_k)) = (E - V_i(t))s_j^1(t - \delta_k)$. Therefore, we obtain state vectors with $n = 4$

components:

$$\dot{x}_1 = \tilde{f}_1(x_1) = f_1(x_1) = f_1([V_1, y_1, z_1, s_1^1]^T) =$$
$$= \begin{bmatrix} y_1 - V_1^3 + bV_1^2 - z_1 + I_1 \\ 1 - 5V_1^2 - y_1 \\ \mu(s(V_1 - x_{rest}) - z_1) \\ \alpha(1 - s_1^1)\frac{1}{1+e^{\nu(V_1 - V_T)}} - \beta s_1^1 \end{bmatrix},$$

$$\dot{x}_i = \tilde{f}_2(x_2) = \tilde{f}_3(x_3) = f_2(x_2) = f_2([V_i, y_i, z_i, s_i^1]^T) =$$
$$= \begin{bmatrix} V_i - \frac{1}{3}V_i^3 + y_i + I_i \\ a + bV_i - cy_i \\ 0 \\ \alpha(1 - s_i^1)\frac{1}{1+e^{\nu(V_i - V_T)}} - \beta s_i^1 \end{bmatrix} \qquad i = 2, 3\ ,$$

2 Step S1: finding the clusters

This method is based on a generalization of the algorithm described in [4, 5] and recently extended in [6]. Generally speaking, a multi-layer network $\aleph$ can be represented through a weighted graph, defined by a vertex set $\mathcal{V} = \{v_1, \ldots, v_N\}$ (representing the oscillators, in our case), an edge set $\mathcal{E}_k$ for each kind of link ($k = 1, \ldots, L$), and a weight set $\mathcal{W}_k$ representing the connection weights for the k-th kind of link.

Two vertices are said to be *adjacent* if there is an edge between them. The *weighted* adjacency matrix A^k embeds the information about the edge set $\mathcal{E}_k$ and the weight set $\mathcal{W}_k$: $A^k_{ij} \in \mathcal{W}_k$ is the (nonzero) weight of the link going from v_j to v_i; $A^k_{ij} = 0$ if there is no edge from v_j to v_i and A^k_{ij} belongs to $\mathcal{W}_k$ otherwise.

In an *undirected* graph, each edge is an unordered pair of vertices $(v_i, v_j) = (v_j, v_i)$; in this case, A^k is a symmetric matrix. On the contrary, in a *directed* graph, each edge is an ordered pair of vertices (v_i, v_j); in this case, A^k is a non-symmetric matrix for at least one value of k.

By a *partition* $\mathcal{C} = \{C_1, C_2, \ldots, C_Q\}$ of a graph, we mean a partition of its vertex set which satisfies the following properties:

$$\mathcal{C} = \left\{ C_i \subset \mathcal{V} : C_i \cap C_j = \emptyset \quad \forall i \neq j, \quad \bigcup_{i=1}^{Q} C_i = \mathcal{V} \right\}$$

where we call C_i the i-th *cluster*, with $i = 1, \ldots, Q$. By assuming that the i-th vertex has a unique label i, we can identify each cluster through the labels of its constituting vertices.

Let N_i be the number of nodes belonging to cluster C_i. Therefore,

$$\sum_{i=1}^{Q} N_i = N.$$

2.1 Equitable partitions and clusters

As stated above, we focus on equitable clusters.[1] A partition $\mathcal{C}$ is *equitable* if it fulfills the following additional condition ($k = 1, \ldots, L$):

$$\sum_{v_a \in C_q} A^k_{ia} = \sum_{v_a \in C_q} A^k_{ja}, \qquad \begin{array}{l} \forall\, v_i, v_j \in C_p \\ \forall\, C_p, C_q \in \mathcal{C} \end{array} . \tag{1}$$

In words, in an equitable partition the nodes belonging to the same cluster receive the same amount of weighted inputs of each type from the other clusters or from the cluster itself.

The clusters of an equitable partition are referred to as ECs [8, 9]. These clusters can be found (in both undirected and directed networks) by extending the algorithm described in [5, 4], as initially proposed in [6].

We assume that the nodes of a partitioned graph can be colored, in such a way that the nodes within the same cluster C_q are given the same color c_q ($q = 1, \ldots, Q$). This corresponds to a *coloring* $\Gamma = \{c_1, \ldots, c_Q\}$ of the nodes.

A coloring Γ of the nodes is *balanced* if the corresponding clustering $\mathcal{C}$ fulfills Eq. (1). A network admits synchronous equitable clusters provided that the clusters correspond to a coloring that is balanced [10, 11, 12, 13]. A *minimal balanced coloring* is a balanced coloring with the minimal number of colors. A coloring $\Gamma' \subseteq \Gamma$ is a *refinement* of a coloring Γ if two nodes that have the same color in Γ' have the same color also in Γ; in other words, Γ' is a smaller set of colors with respect to Γ.

The *input-driven refinement* Γ' of a coloring Γ is obtained as follows. Consider all nodes that have color c_j in Γ, with $j \in \{1, \ldots, Q\}$. These nodes are assigned the same color if they receive the same number of inputs of kind k from all nodes with the same color in Γ.

The algorithm for computing the minimal balanced coloring is articulated in the following three steps:

1. color all nodes of the same type with the same color;

2. replace the current coloring C with the input driven refinement Γ' of the current coloring;

3. repeat step 2 until no new refinement is obtained, i.e., no new color is added to Γ.

This algorithm allows obtaining the minimal balanced coloring of a given network in a finite number of steps. The proof of this statement is analogous to that proposed in [4, 5], provided that the above generalized definitions are used: therefore, it is not reported here.

[1] *Orbital clusters* are defined in terms of the network symmetries [7], whereas *equitable clusters* depend on the inputs each node receives [8, 9]. By definition, orbital clusters are a particular case of equitable clusters.

2.2 Examples

Fig. 2 shows a simple example, used as reference to illustrate some of the main concepts and definitions introduced above, of a network with $N = 7$ nodes of $M = 2$ kinds (denoted by solid and dashed circles), which are connected through $L = 3$ different kinds of links, denoted by a square, a dot and a diamond. Arrows mark directed edges; if an edge has no arrow, it is undirected. Figure 2(a), in particular, evidences the network layers, where each layer $1, \ldots, M$ encompasses nodes of the same kind, whereas Fig. 2(b) shows the corresponding graph.

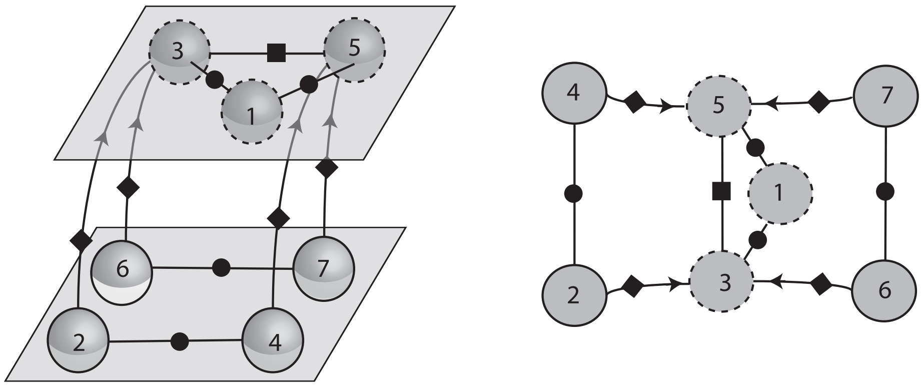

(a) (b)

Figure 2: A 7-node 2-layer network (a) and its graph (b).

Fig. 3 shows step 1 (panel a) of the algorithm for computing the minimal balanced coloring and the obtained minimal balanced coloring (panel b) for the multi-layer network of Fig. 2.

By assuming equal link weights A_{ij}^k, the directed network shown in 3c is color-balanced, as: each red node receives one diamond-input from green and yellow nodes, one dot-input from gray nodes and one square-input from red nodes; each green (yellow) node gets a dot-input from the green (yellow) nodes.

The network shown in Fig. 3b has a minimal balanced coloring, whereas Fig. 3c shows a color-balanced network without minimal balanced coloring.

Fig. 3b is a refinement of Fig. 3c, as nodes 2 and 4 share the same color in both figures.

Fig. 3b is an input-driven refinement of Fig. 3c.

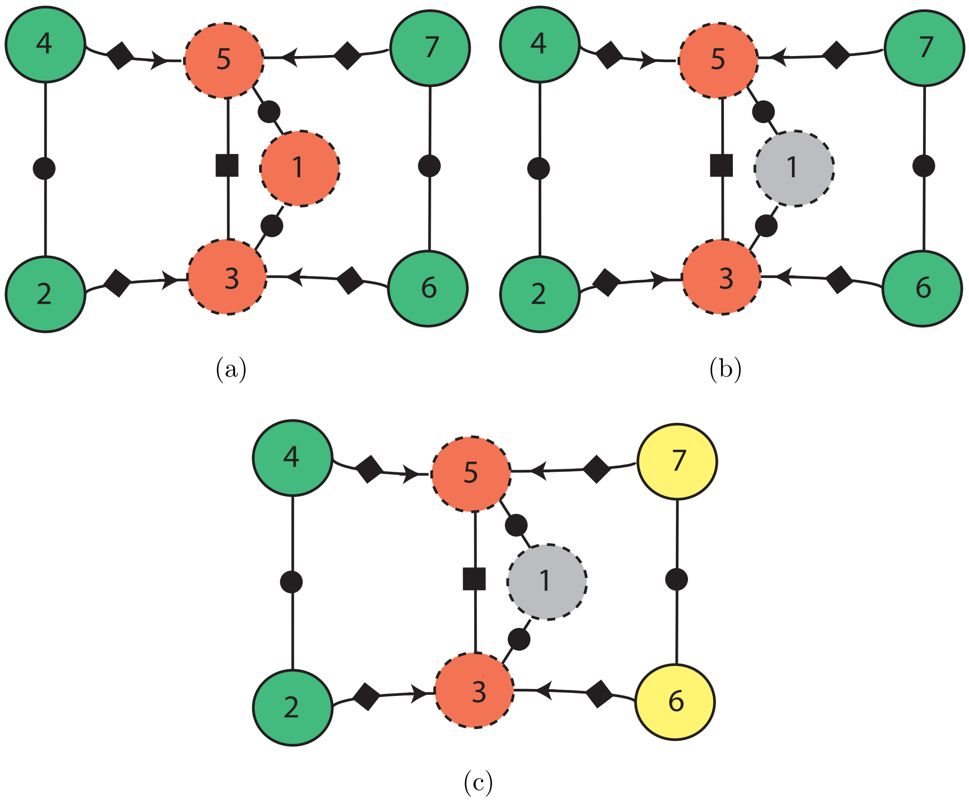

Figure 3: Examples of coloring for the multi-layer network of Fig. 2: step 1 of the algorithm (a), minimal balanced coloring (b), coloring example used to illustrate the concept of input-driven refinement (c).

3 Step S2: from clustered network to quotient network

To start with, we summarize some further general concepts about networks, graphs, symmetries and partitions.

A *permutation* of the vertices of a graph $\aleph$ is a bijection $\pi : \mathcal{V} \to \mathcal{V}$ which can be thought of as a re-shuffling of the vertices, without altering the elements of $\mathcal{V}$. Each permutation can be represented by a permutation matrix Π, whose entries are $\Pi_{ij} = 1$ if $\pi(v_j) = v_i$ and $\Pi_{ij} = 0$ otherwise. Notice that Π is orthonormal by definition, as $\Pi\Pi^T$ is an identity matrix.

A *symmetry* of a graph is a permutation such that the edge set $\mathcal{E}_k$ and the connection weights $\mathcal{W}_k$ remain unchanged for any k after the permutation. The set of these symmetries is a group $\mathcal{G}$ called the *automorphism group*.

Analogously, the symmetries of a network corresponding to the graph $\aleph$ and described by Eq. (1) in the paper belong to the group $\mathcal{G}$. Each element g of this group can be

described in turn by a permutation matrix Π, which re-arranges the nodes in a way that leaves Eq. (1) in the paper unchanged, that is, $\Pi : \Pi A^k = A^k \Pi$ for any k.

The *orbits* of the symmetry group $\mathcal{G}$ are disjoint sets of nodes that permute among one another under the action of all the symmetries $\{\Pi\}$ of the group [14].

The coarsest *orbital* partition $\mathcal{C}$ contains the set of orbits induced by the automorphism group $\mathcal{G}$.

Henceforth, the clusters of an orbital partition are referred to as *orbital clusters* (OCs) **[15, 16]**. As Eq. (1) in the paper is essentially unchanged by the permutations within an orbital cluster, the dynamics of the nodes in each cluster can be equal, which is exact synchronization [7].

Equitable clusters are the most general case: by definition, orbital clusters are equitable, whereas the converse is false, in general.

Algorithms are available to find the group $\mathcal{G}$ and the Q OCs in both undirected and directed networks [17, 18], provided that the networks are homogeneous, i.e., all nodes/neurons are described by the same model. The same algorithms apply also to the general heterogeneous case, by representing the multi-layer network through a colored multi-partite graph. Therefore, the symmetries of a graph which defines the automorphism group $\mathcal{G}$ (and therefore the OCs) must be permutations which swap only nodes of the same kind.

As an example to which apply the above concepts, we consider the network shown in Fig. 2(b) and the matrix

$$\begin{bmatrix} 1 & 0 & 0 & 0 & 0 & 0 & 0 \\ 0 & 0 & 0 & 1 & 0 & 0 & 0 \\ 0 & 0 & 0 & 0 & 1 & 0 & 0 \\ 0 & 1 & 0 & 0 & 0 & 0 & 0 \\ 0 & 0 & 1 & 0 & 0 & 0 & 0 \\ 0 & 0 & 0 & 0 & 0 & 0 & 1 \\ 0 & 0 & 0 & 0 & 0 & 1 & 0 \end{bmatrix}, \tag{2}$$

which defines a permutation $\bar{\pi}$ that exchanges the couples of nodes $[2, 4]$, $[3, 5]$ and $[6, 7]$.

The permutation $\bar{\pi}$ is also a symmetry of the network in Fig. 2(b), since the network after the permutation holds its original structure. The symmetries of the network in Fig. 2 must not swap nodes 1 and 2 since they are of different kinds.

The orbital partition corresponding to the symmetry $\bar{\pi}$ contains four OCs, which are $C_1 = [1]$, $C_2 = [2, 4]$, $C_3 = [3, 5]$ and $C_4 = [6, 7]$. More in general, by applying all the symmetries of this network, we obtain that node 3 can be swapped only with node 5, node 2 can be swapped with nodes 4, 6, 7, while node 1 cannot be swapped with any of the other nodes of the network. This allow us to find the lowest number (three) of OCs, which are $C_1 = [1]$, $C_2 = [2, 4, 6, 7]$ and $C_3 = [3, 5]$.

The quotient network corresponding to the clustering in Fig. 3(b) is shown in Fig. 4: notice that the nodes in each cluster are now represented as a single node.

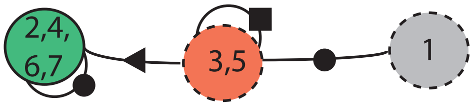

Figure 4: Graph of the quotient network (with $Q = 3$ nodes) corresponding to the example network of Fig. 2, clustered as shown in Fig. 3(b).

The projector operator for the example network of Fig. 2 is

$$P^{\mathcal{C}} = \begin{bmatrix} 1 & 0 & 0 \\ 0 & 0 & 1 \\ 0 & 1 & 0 \\ 0 & 0 & 1 \\ 0 & 1 & 0 \\ 0 & 0 & 1 \\ 0 & 0 & 1 \end{bmatrix}.$$

We point out that all nodes within a cluster are necessarily of the same kind, i.e., the corresponding isolated nodes are described by the same model.

We collect all state trajectories in the vector $x(t) = [x_1^T(t), x_2^T(t), \ldots, x_N^T(t)]^T$. As it is possible for all the nodes within a cluster to synchronize, we define the q-th cluster state: $s_q(t) = x_i(t)$ for all i in cluster C_q. Correspondingly, the network can produce Q distinct synchronized motions $\{s_1(t), s_2(t), \ldots, s_Q(t)\}$, one per cluster. We collect them in the vector $s(t) = [s_1^T(t), s_2^T(t), \ldots, s_Q^T(t)]^T$.

Remarks about the projection operator $P^{\mathcal{C}}$:

- the vector field f_q of each node belonging to cluster C_q is mapped onto itself;
- for any q, $\left(P^{\mathcal{C}}\right)^T x$ allows extracting from x all the state trajectories $x_i(t)$ corresponding to C_q;
- $\left(P^{\mathcal{C}}\right)^T P^{\mathcal{C}}$ is a diagonal matrix whose entry qq is the number of nodes belonging to cluster C_q;
- by pre-multiplying the vector x by the pseudo-inverse matrix of $P^{\mathcal{C}}$, we compute the vector s, where
$$s_q(t) = \frac{1}{N_q} \sum_{i \in C_q} x_i(t).$$

Once the clustering $\mathcal{C} = \{C_1, \ldots, C_Q\}$ is defined (step S1), a *quotient network* [11, 19] can be constructed whose nodes correspond to the synchronous clusters of the original network $\aleph$. The dynamics on the quotient network can be described as the dynamics of the original ODE on $\aleph$ restricted to a flow-invariant subspace. This is the *synchronous*

subspace associated with $\mathcal{C}$, which consists of all points in state space whose coordinates are identical for nodes that are mapped together under a projector operator (say, $P^{\mathcal{C}}$) on $\mathcal{C}$.

We introduce the projector operator on $\mathcal{C}$ as

$$P^{\mathcal{C}} = \left[e_{C_1} \quad e_{C_2} \quad \ldots \quad e_{C_Q} \right],$$

where the column vectors e_{C_q}, $q = 1, \ldots, Q$ span the synchronous subspace ($e_{C_q,i} = 1$ if $i \in C_q$, 0 otherwise). The projector $P^{\mathcal{C}}$ defines the transformation from the quotient network to the corresponding N-nodes network: $x(t) = P^{\mathcal{C}} s(t)$. Thus the quotient system dynamics is governed by the following equations

$$\dot{s}_q = f_q(s_q) + \sum_{k=1}^{L} \sigma^k \sum_{p=1}^{Q} R^k_{pq} h^k(s_q(t), s_p(t - \delta_k)), \tag{3}$$

where f_q is the vector field of each node belonging to cluster C_q (i.e., $f_q = \tilde{f}_i$ for any $i \in C_q$) and $R^k = \left(\left(P^{\mathcal{C}}\right)^T P^{\mathcal{C}} \right)^{-1} \left(P^{\mathcal{C}}\right)^T A^k P^{\mathcal{C}}$ is the Q-dimensional quotient network adjacency matrix [8].

Notice that the presence of different neuron models determines different expressions for the vector fields f_q in Eq. (3).

4 Structure of matrix T for directed networks

The matrix T converts the node coordinate system to the IRR coordinate system, thus evidencing the interdependencies among perturbation components.

The first Q rows of T are the vectors whose span generates the synchronous manifold of the transformed linearized system. For each one of these rows: entry i of of row j is equal to $\frac{1}{\sqrt{N_j}}$ (remember that N_j is the number of nodes in cluster C_j) if node i is in cluster C_j and 0 otherwise.

The remaining rows span the transverse manifold of the transformed linearized system. More in detail, N_j rows of T correspond to cluster C_j: one row in the upper part of T, spanning the synchronization manifold and $N_j - 1$ rows in the lower part of T, spanning the transverse manifold.

For directed networks where the directed connections originate from or go to trivial clusters only, matrix T can be constructed as follows:

- We find the clusters on the complete network.
- We know that trivial clusters contain only one node and there is no synchronization stability to analyze. Therefore, we remove all directed connections and maintain the original clustering, thus obtaining a network colored like the original one, but undirected.

- For this network, the IRR can be found through the method described in this paper.

For directed networks where each cluster contains at most two nodes, matrix T can be constructed as follows. Since each cluster contains at most two nodes, each of the first Q rows of matrix T contains at most two non-null components. Therefore, there is only one direction orthogonal to each of these rows. As a consequence, matrix T is unique and defines the orthonormal IRR change of coordinates.

5 Properties of the matrices used to describe the perturbation dynamics

- J_q is a diagonal matrix.

  Proof. This is a consequence of the structure of matrix T, each row of which depends on only one cluster by construction. All rows of T are orthonormal. Consider the product $\tilde{J}_q = TE_{C_q}$: since $E_{C_q,ij} = 1$ only if $j = i \in C_q$, $\tilde{J}_{q,ij} = 0$ $\forall i$ if $j \notin C_q$, $\tilde{J}_{q,ij} = T_{ij}$ $\forall i$ if $j \in C_q$; in words, the product TE_{C_q} keeps only the T rows which depend on cluster C_q and sets to 0 all the entries of T that are not in the columns related to C_q. Therefore, $J_q = \tilde{J}_q T^T$ is a matrix with diagonal entries $J_{q,ii} = 1$ only if $i \in C_q$. □

- for undirected networks, B^k is block diagonal with at least two blocks, one of size $Q \times Q$ and the other of size $(N - Q) \times (N - Q)$ (see [7] for the proof).

- for directed networks, B^k is block upper-triangular with at least two blocks, one of size $Q \times Q$ and the other of size $(N - Q) \times (N - Q)$

  Proof. We first recall that the cluster synchronization manifold is A^k-invariant [20]: this means that the image through A^k of any vector that lies in the cluster-synchronous subspace (any linear combination of the first Q rows of the matrix T) lies in the space spanned by the first Q rows of the matrix T. Since

$$B^k = TA^kT^T \longrightarrow A^kT^T = T^TB^k = \left((B^k)^TT\right)^T,$$

  and recalling that the remaining $N - Q$ rows of T are orthogonal to the first Q, we obtain that $(B^k)^T$ is 0 in all the last $N - Q$ columns of the first Q rows. This means that B^k is block upper-triangular. □

- $J_qB^kJ_p$ has the same structure of B^k.

Proof. As shown above, J_q is a diagonal matrix, and therefore can be interpreted as a matrix with the same structure of B_k. Therefore, their product is still a matrix (block diagonal for undirected networks, block upper-triangular for directed networks) with the same structure of B_k. □

6 Matrices for the macaque cerebral cortex

Fig. 5 shows the structure of the matrices T (left) and B^k (right) for the swim CPG network.

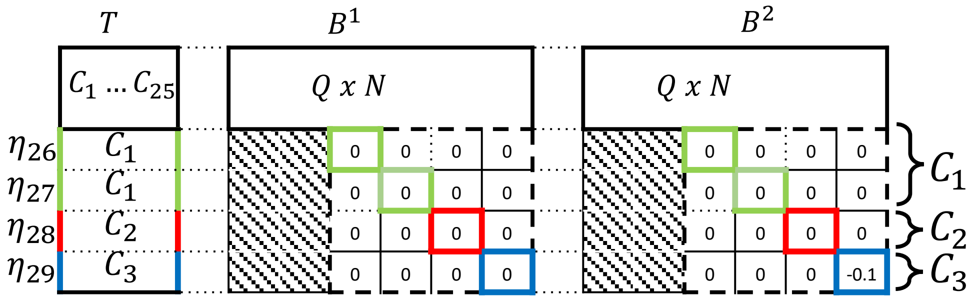

Figure 5: Structure of the matrices T, B^1 **and** B^2 for the macaque cerebral cortex. The gray blocks correspond to 0 entries.

The gray blocks correspond to 0 entries. In the block upper-triangular matrices B^k, the first Q rows are related to the perturbation dynamics along the synchronous manifold. Each white sub-block in the lower-right $(N-Q)\times(N-Q)$ sub-matrix B^k_{N-Q} describes the perturbation dynamics transverse to the synchronous manifold, thus is associated with loss of synchronization, either transient or permanent depending on the cluster stability.

If we analyze the matrices B^k (related to the k-th connection type), we can see that:

- B^1_{N-Q} (related to undelayed chemical excitatory synapses) has only zero entries; this implies that for the network with only these synapses, the dynamics of each perturbation component η_k depends only on η_k through the term ρ_1 in Eqs. (2) and (4) in the paper;

- B^2_{N-Q} (related to delayed chemical excitatory synapses) has one 1×1 sub-block (with blue borders) related to cluster C_3; this means that for the network with only the delayed chemical excitatory synapses the dynamics of the perturbation component η_{29} depends on η_{29} through both ρ_1 and ρ_2 in Eqs. (2) and (4) in the paper.

In summary, if we consider the whole network, with all kinds of synapses, the three clusters C_1, C_2, C_3 turn out to be not intertwined.

Datasets

Supplementary Dataset 1 (dendronotus.xlsx): Matrices for the swim CPG example.
Supplementary Dataset 2 (macaque.xlsx): Matrices for the macaque example.

References

[1] Hindmarsh, J. L. & Rose, R. A model of neuronal bursting using three coupled first order differential equations. *Proceedings of the Royal society of London. Series B. Biological sciences* **221**, 87–102 (1984).

[2] FitzHugh, R. Impulses and physiological states in theoretical models of nerve membrane. *Biophysical journal* **1**, 445 (1961).

[3] Jalil, S., Belykh, I. & Shilnikov, A. Spikes matter for phase-locked bursting in inhibitory neurons. *Physical Review E* **85**, 036214 (2012).

[4] McKay, B. D. *et al. Practical graph isomorphism* (Department of Computer Science, Vanderbilt University Tennessee, USA, 1981).

[5] Belykh, I. & Hasler, M. Mesoscale and clusters of synchrony in networks of bursting neurons. *Chaos: An Interdisciplinary Journal of Nonlinear Science* **21**, 016106 (2011).

[6] Lodi, M., Della Rossa, F., Sorrentino, F. & Storace, M. An algorithm for finding equitable clusters in multi-layer networks. In *2020 IEEE International Symposium on Circuits and Systems (ISCAS)*, 1–4 (IEEE, 2020).

[7] Pecora, L. M., Sorrentino, F., Hagerstrom, A. M., Murphy, T. E. & Roy, R. Cluster synchronization and isolated desynchronization in complex networks with symmetries. *Nature communications* **5**, 4079 (2014).

[8] Schaub, M. T. *et al.* Graph partitions and cluster synchronization in networks of oscillators. *Chaos: An Interdisciplinary Journal of Nonlinear Science* **26**, 094821 (2016).

[9] Siddique, A. B., Pecora, L., Hart, J. D. & Sorrentino, F. Symmetry-and input-cluster synchronization in networks. *Physical Review E* **97**, 042217 (2018).

[10] Wang, Y. & Golubitsky, M. Two-colour patterns of synchrony in lattice dynamical systems. *Nonlinearity* **18**, 631 (2004).

[11] Golubitsky, M., Stewart, I. & Török, A. Patterns of synchrony in coupled cell networks with multiple arrows. *SIAM Journal on Applied Dynamical Systems* **4**, 78–100 (2005).

[12] Golubitsky, M. & Stewart, I. Nonlinear dynamics of networks: the groupoid formalism. *Bulletin of the american mathematical society* **43**, 305–364 (2006).

[13] Aguiar, M. A. & Dias, A. P. S. Synchronization and equitable partitions in weighted networks. *Chaos: An Interdisciplinary Journal of Nonlinear Science* **28**, 073105 (2018).

[14] Golubitsky, M., Stewart, I. & Schaeffer, D. G. *Singularities and groups in bifurcation theory*, vol. 2 (Springer Science & Business Media, 2012).

[15] Tinkham, M. *Group theory and quantum mechanics* (Courier Corporation, 2003).

[16] Gallian, J. *Contemporary abstract algebra* (Nelson Education, 2012).

[17] Stein, W. & Joyner, D. SAGE: System for algebra and geometry experimentation. *ACM Sigsam Bulletin* **39**, 61–64 (2005).

[18] Seress, Á. *Permutation group algorithms*, vol. 152 (Cambridge University Press, 2003).

[19] Aguiar, M. A., Dias, A. P. S., Golubitsky, M. & Maria da Conceição, A. L. Bifurcations from regular quotient networks: a first insight. *Physica D: Nonlinear Phenomena* **238**, 137–155 (2009).

[20] Schaub, M. T. *et al.* Graph partitions and cluster synchronization in networks of oscillators. *Chaos: An Interdisciplinary Journal of Nonlinear Science* **26**, 094821 (2016).
